# Transcription factor ZmEREB97 regulates nitrate uptake in maize (*Zea mays*) roots

**DOI:** 10.1093/plphys/kiae277

**Published:** 2024-05-14

**Authors:** Qi Wu, Jinyan Xu, Yingdi Zhao, Yuancong Wang, Ling Zhou, Lihua Ning, Sergey Shabala, Han Zhao

**Affiliations:** Provincial Key Laboratory of Agrobiology, Jiangsu Academy of Agricultural Sciences, Nanjing 210014, China; Institute of Crop Germplasm and Biotechnology, Jiangsu Academy of Agricultural Sciences, Nanjing 210014, China; Provincial Key Laboratory of Agrobiology, Jiangsu Academy of Agricultural Sciences, Nanjing 210014, China; Institute of Crop Germplasm and Biotechnology, Jiangsu Academy of Agricultural Sciences, Nanjing 210014, China; Institute of Crop Germplasm and Biotechnology, Jiangsu Academy of Agricultural Sciences, Nanjing 210014, China; Provincial Key Laboratory of Agrobiology, Jiangsu Academy of Agricultural Sciences, Nanjing 210014, China; Institute of Crop Germplasm and Biotechnology, Jiangsu Academy of Agricultural Sciences, Nanjing 210014, China; Provincial Key Laboratory of Agrobiology, Jiangsu Academy of Agricultural Sciences, Nanjing 210014, China; Institute of Crop Germplasm and Biotechnology, Jiangsu Academy of Agricultural Sciences, Nanjing 210014, China; Provincial Key Laboratory of Agrobiology, Jiangsu Academy of Agricultural Sciences, Nanjing 210014, China; Institute of Crop Germplasm and Biotechnology, Jiangsu Academy of Agricultural Sciences, Nanjing 210014, China; School of Biological Science, University of Western Australia, Crawley, WA 6009, Australia; Department of Horticulture and International Research Centre for Environmental Membrane Biology, Foshan University, Foshan 528000, China; Provincial Key Laboratory of Agrobiology, Jiangsu Academy of Agricultural Sciences, Nanjing 210014, China; Institute of Crop Germplasm and Biotechnology, Jiangsu Academy of Agricultural Sciences, Nanjing 210014, China; Key Laboratory of Germplasm Innovation in Downstream of Huaihe River, Jiangsu Academy of Agricultural Science, Nanjing 210014, China

## Abstract

Maize (*Zea mays* L.) has very strong requirements for nitrogen. However, the molecular mechanisms underlying the regulations of nitrogen uptake and translocation in this species are not fully understood. Here, we report that an APETALA2/ETHYLENE RESPONSE FACTOR (AP2/ERF) transcription factor ZmEREB97 functions as an important regulator in the N signaling network in maize. Predominantly expressed and accumulated in main root and lateral root primordia, ZmEREB97 rapidly responded to nitrate treatment. By overlapping the analyses of differentially expressed genes and conducting a DAP-seq assay, we identified 1,446 potential target genes of ZmEREB97. Among these, 764 genes were coregulated in 2 lines of *zmereb97* mutants. Loss of function of ZmEREB97 substantially weakened plant growth under both hydroponic and soil conditions. Physiological characterization of *zmereb97* mutant plants demonstrated that reduced biomass and grain yield were both associated with reduced nitrate influx, decreased nitrate content, and less N accumulation. We further demonstrated that ZmEREB97 directly targets and regulates the expression of 6 *ZmNRT* genes by binding to the GCC-box-related sequences in gene promoters. Collectively, these data suggest that ZmEREB97 is a major positive regulator of the nitrate response and that it plays an important role in optimizing nitrate uptake, offering a target for improvement of nitrogen use efficiency in crops.

## Teaser

An ethylene response transcription factor plays an important role in the nitrogen signaling network and in optimizing nitrate uptake in maize.

## Introduction

Nitrogen (N) is an essential macronutrient for plants, controlling almost every aspect of plant growth and development. Owing to the wide abundance of a synthetic N application in farmland, the yield of crops was dramatically increased in the past 50 yr (Han et al. 2015). This increase, however, comes with 2 caveats. The first one is that modern crop varieties become less and less responsive to additional N supply (Fischer et al. 2009). The second concern is that excessive use of nitrogenous fertilizers is expensive and leads to considerable negative impacts on the ecosystems due to eutrophication (Vitousek et al. 2009; Good and Beatty 2011). New solutions are therefore needed to simultaneously increase yield while maintaining, or preferably decreasing, the amounts of applied N, to reduce the environmental footprint and maximize N use efficiency (NUE) of crops.

Maize (*Zea mays* L.) is the crop species with the highest global annual yield (Ort and Long 2014; Hochholdinger et al. 2017). N is a major contributor to the high yield of maize, prompting high demand for N fertilizers. Meanwhile, balancing sufficient N supply for maize yield and preventing leaching of N fertilizers in natural and agricultural ecosystems are highly challenging (Robertson and Vitousek 2009). An increased understanding of how maize plants respond to N supply and dissecting the downstream components of N signaling pathways is essential in the refinement of strategies to improve NUE.

Plant NUE is inherently complex, since it involves N sensing, uptake, transportation, assimilation, and remobilization and is governed by multiple interacting genetic and environmental factors (Xu et al. 2012; Krapp 2015). As a result, N sensing followed by uptake is the initial step in N cycle and represents the foundation for NUE. N uptake is mediated by the nitrate transporters (NRTs), which have been most thoroughly characterized in Arabidopsis (*Arabidopsis thaliana*). The Arabidopsis genome encodes at least 67 NRTs, including 53 *NRT1* genes, 7 *NRT2* genes, and 7 chloride channel genes (O’Brien et al. 2016). In rice, there are 5 known NRTs—e.g. OsNRT1.1A (Wang et al. 2018), OsNRT1.1B (Hu et al. 2015), OsNRT2.1 (Katayama et al. 2009), OsNRT2.2 (Feng et al. 2011; Yan et al. 2011), and OsNRT2.3a (Fan et al. 2016). In maize, 2 high-affinity NRTs ZmNRT2.1 and ZmNRT2.2 and 2 low-affinity NRTs ZmNPF6.4 and ZmNPF6.6 have been characterized and linked with nitrate uptake (Trevisan et al. 2008; Wen et al. 2017). However, little is known about how the expression of these transporter genes is regulated in response to N supply in maize.

Transcription factors (TFs) often act as master switches in plant regulatory networks (Xuan et al. 2017; Plett et al. 2018). ANR1 is an early discovered TF known to regulate systemic nitrate repression and participate in the signaling pathway of NRT1.1 in Arabidopsis (Zhang and Forde 1998; Gan et al. 2005). Other TFs were shown in controlling primary nitrate-responsive genes such as the NRT genes (*NRT1.1*, *NRT2.1*, *NRT2.2*), the nitrate reductase (NR) genes (*NIA1*, *NIA2*), and the nitrite reductase gene (*NIR*). This includes NLP6 (Konishi and Yanagisawa 2013), NLP7 (Castaings et al. 2009; Marchive et al. 2013), LBD37/38/39 (Rubin et al. 2009), TCP20 (Guan et al. 2014, 2017), SPL9 (Krouk et al. 2010), and NIGT1/HRS1s (Kiba et al. 2018). In Arabidopsis, these TFs bind to the promoter of *NRT1.1*, *NRT2.1*, *NRT2.2*, *NIA1*, or *NIA2* genes and regulate their expression (Vidal et al. 2015). Recently, key TFs such as OsTCP19, OsNGR5, OsNAC42, and OsGRF4 were reported in rice. OsTCP19 can modulate the expression of N-responsive genes, and overexpression of *OsTCP19* gene confers a higher N absorption ability (Liu et al. 2021). An increase in the transcription level of a TF gene, *OsNGR5*, results in an increase in rice yield and NUE for the same level of N supply (Wu et al. 2020). A rice N transporter OsNPF6.1^HapB^, trans-activated by a NUE-related TF OsNAC42, enhances nitrate uptake (Tang et al. 2019). OsGRF4 promotes and integrates N assimilation, carbon fixation, and growth (Li et al. 2018). In maize, ZmNLP5 was found to be one of the key TFs in the molecular network for mediating N signaling and metabolism. A natural loss-of-function allele of *ZmNLP5* conferred less N accumulation in the ear leaves and seed kernels resembling (Ge et al. 2020). However, the molecular mechanisms underlying transcriptional regulation of N uptake in maize remain largely unknown.

Here, using the combined approaches of gene coexpression network analysis, promoter sequence motif prediction, and yeast one hybrid (Y1H) screening, we identified a TF, ZmEREB97, that belongs to the AP2/ERF family. The AP2/ERF family is one of the largest plant TF families that consist of many subfamilies such as AP2, ERF (also known as ethylene-responsive element binding proteins, EREBPs), dehydration-responsive element binding protein (DREB), and RAV (related to ABI3/VP1) (Chandler 2018). Members of AP2/ERF TFs have emerged as regulators for plant developmental and abiotic stress responses (Licausi et al. 2013). The characteristic DNA-binding domain of AP2/ERF, which consists of a sheet with 3 β-strands followed by an α-helix, recognizes specific cis-regulatory elements, including the GCC-box (Shoji and Yuan 2021). The combined physiological, molecular, and genetic analyses indicate that ZmEREB97 concomitantly mediates N influx by regulating several NRT genes. The loss of *ZmEREB97* gene resulted in downregulation of nitrate uptake and accumulation suggesting its critical role in optimizing N acquisition and utilization.

## Results

### Coexpression network analysis with WGCNA

To predict the conserved motifs in nitrate-related genes, a gene coexpression network was performed using 2 representative inbred maize lines B73 (a stiff stalk [SS] inbred) and Mo17 (a non-stiff stalk [NSS] inbred; Mikel and Dudley 2006). To identify the nitrate sensitivity between B73 and Mo17, we designed a “recovery test” under 2 different N conditions. As shown in Supplementary Fig. S1, both B73 and Mo17 showed symptoms of nitrate starvation under deficient nitrate conditions, with no significant differences observed in Soil Plant Analysis Development (SPAD) values, nitrate content, and total N content. However, after being supplied with sufficient N (10 mm KNO_3_) for 10 d, B73 exhibited a substantial increase in SPAD values (88.1%), nitrate content (72.2%), and total N content (90.4%), which were significantly higher than the corresponding values in Mo17 (53.7%, 65.4%, and 81.7%, respectively). These results suggest that B73 has a faster recovery rate from N deficiency as compared to Mo17, indicating that it is a more nitrate-sensitive inbred maize line.

Firstly, MapMan software was used to visualize the biological processes affected by nitrate treatment (Thimm et al. 2004). As shown in Supplementary Fig. S2 and Data Set 1, notable changes were observed in many metabolic processes in root tissues of B73 and Mo17 treated with 15 mm KNO_3_ at 0.5- and 8-h timepoints, as compared with 15 mm KCl. These included minor CHO, light reactions, photorespiration, TCA and OPP cycles, and amino acid and N metabolism. In addition, carbon and carbohydrate metabolisms were also affected by nitrate treatment in this study, suggesting their close relationship with N metabolism.

**Figure 1.**
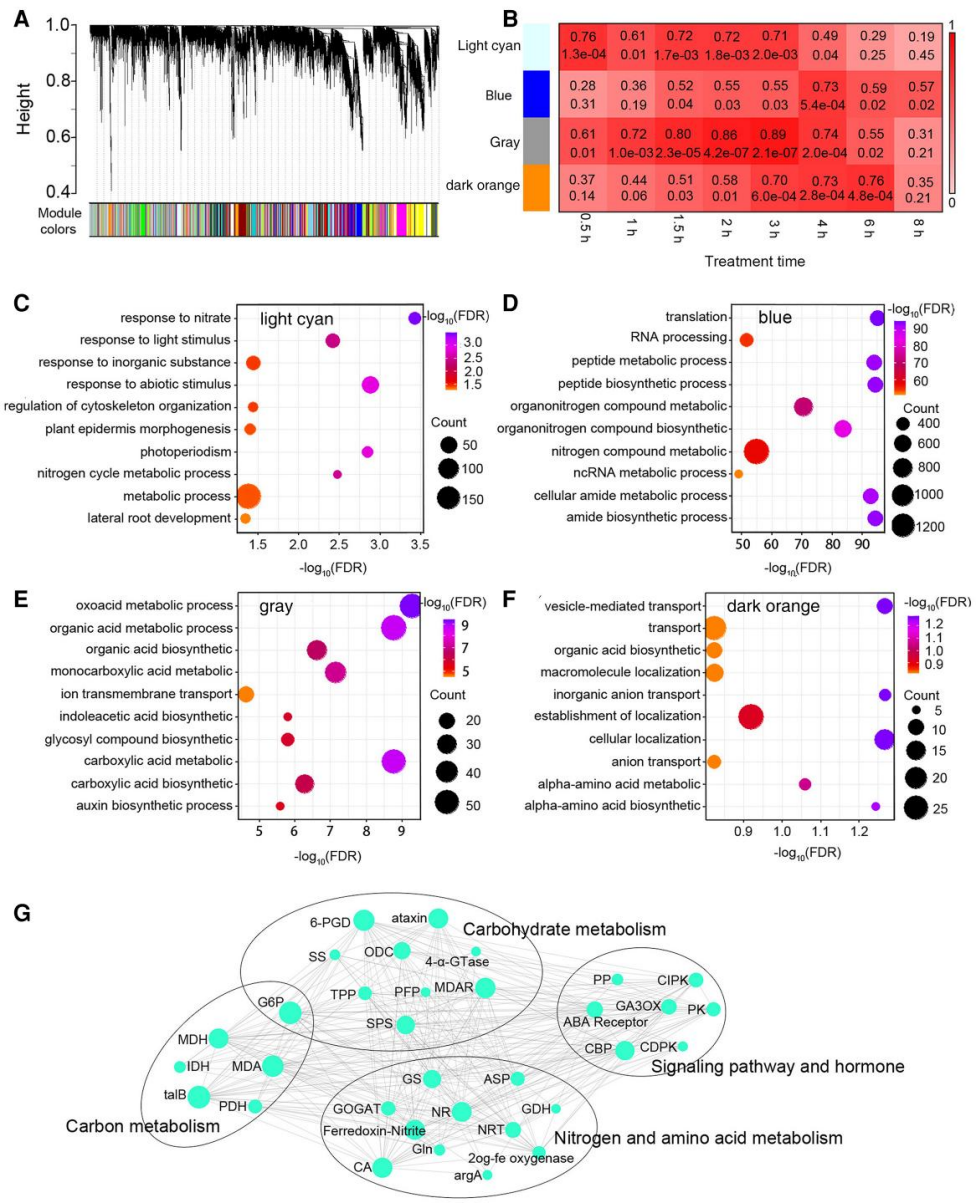
Network and GO enrichment analysis of maize roots’ response to nitrate supply. **A)** Hierarchical cluster tree showing coexpression modules identified using WGCNA. Modules correspond to branches and are labeled by colors. **B)** Matrix with the modules and time points after nitrate supply relationships. Each row corresponds to a module eigengene and column to a time point. Each cell contains the corresponding correlation coefficient (up) and *P*-value (down). The table is color-coded by correlation according to the color legend. **C to F)** GO enrichment analyses showing N-related processes are enriched in four coexpression modules. **G)** A schematic diagram of representative highly connected genes and network of the four coexpression modules, showing high correlation with N, carbon, carbohydrate metabolism, signaling pathway, and hormone.

We then applied the WGCNA on RNA sequencing (RNA-Seq) data of 36 root tissue samples to construct the consensus network of B73 and Mo17. As a result, 26 modules were identified and labeled by different colors in the hierarchical clustering dendrogram (Fig. 1A), with each module containing between 79 and 3,434 genes (Supplementary Data Set S1). By relating consensus modules to B73 set-specific modules, it was shown that the module structure of the B73 expression data was slightly different from the Mo17 expression data (Supplementary Figs. S3 and S4). To determine if any of the genes in the 26 modules was associated with nitrate treatment time, we tested the association of module eigengenes (the first principal component of a given module that can be considered as a representative of the gene expression profiles in a module) with the nitrate treatment time. First, the relationships between modules and nitrate treatment time were calculated in the B73 and Mo17 datasets, respectively. Then, the consensus eigengene networks in the B73 and Mo17 datasets were compared, and 4 consensus modules “dark orange,” “gray,” “blue,” and “light cyan” were identified which showed high correlation to the time points after nitrate supply (corresponding correlation > 0.7, *P* < 0.01; Fig. 1B, Supplementary Data Set 1). The module membership (MM; the correlation of the module eigengene and the gene expression profile) and gene significance (GS; quantifies an association of individual genes with nitrate treatment time points) plots for the 4 modules showed that MM and GS are highly correlated, indicating that genes significantly associated with nitrate treatment time were also the most important elements in the modules (Supplementary Fig. S3 and Data Set 1).

Finally, gene ontology (GO) enrichment analysis on the 4 modules and the top 10 most enriched biological processes in “dark orange”, “gray”, “blue,” and “light cyan” modules were conducted. The results showed that N metabolic processes were enriched in the “blue” and “light cyan” modules; photoperiodic processes were enriched in the “light cyan” module; carboxylic acid metabolic/biosynthetic processes and hormone biosynthetic process were enriched in the “gray” module; and amide metabolic/biosynthetic processes were enriched in the “blue” module (Fig. 1, C to F, Supplementary Data Set 1). Network visualization using the set of genes from these biological processes in the 4 modules showed that these genes were highly connected, and each gene had more than 2 edges to interact with other genes (Fig. 1G, Supplementary Data Set 1).

Collectively, these results show that 4 consensus modules that are conserved in 2 different inbred lines B73 and Mo17 are closely related to N. Exploration of the genes with similar expression patterns in the 4 modules and mining their conserved regulatory elements may shed light on more knowledge of maize response to N supplementation.

### GCC-box is enriched in the promoter region of genes in the 4 N-related modules

**Figure 2.**
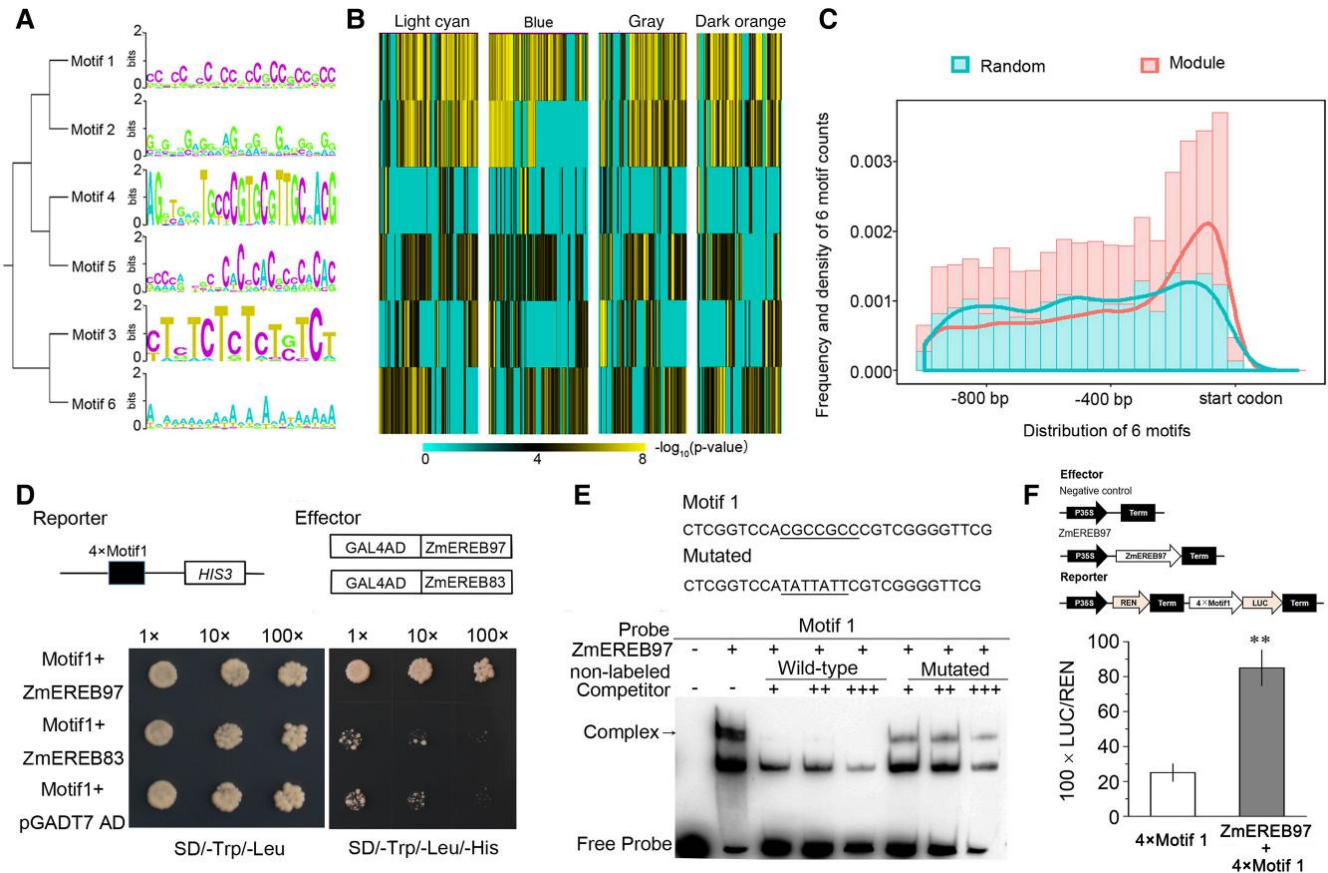
Identification of ZmEREB97. **A to C)** De novo motif discovery of 6 motifs and their enrichment in the 4 N-related modules. **A)** Relationship tree and sequence logos for 6 sequence motifs. **B)** The enrichment of 6 motifs in the promoter of genes from the 4 N-related modules. **C)** Frequency distribution histogram and density map showing the position bias of 6 motifs in the promoter of genes from 4 modules and 500 random genes. **D)** Y1H assay. Yeast harboring the 4×Motif 1–HIS3 reporter gene was transformed with expression vectors for the GAL4 activation domain fused to the ZmEREB97 and ZmEREB83 (control) and grown on control (-Trp/-Leu) and selection medium (-His/-Trp/-Leu + 60 mm 3-AT). **E)** EMSA with 6×His–ZmEREB97 fusion protein using Motif 1 as a probe. Nonlabeled WT and mutated Motif 1 were used as competitor. **F)** Transcriptional activity assays of ZmEREB97. The 35S::REN-4×Motif 1::LUC reporter construct was transiently expressed in *N. benthamiana* leaves together with the negative control vector and the 35S::ZmEREB97 effector vector. The reporter construct contains 2 reporter genes, the LUC and the REN. 4×Motif 1 activities are given as fLUC/rLUC activity ratios. The bars represent means ± Sd (*n* = 3). Two-tailed Student's *t* test (*n* = 3 collective samples each containing 3 biological replicates) was used to test the significance. **P < 0.01.

The putative promoter sequences (1000-bp upstream and 100-bp downstream of the start codon) of the genes in each module were subjected to MEME Suite program for de novo motif discovery. A total of 6 motifs obtained from the 4 modules (*E* < 0.05) can be classified into 2 types according to their matrix (Fig. 2A, Supplementary Data Set 2), and 4 of them were significantly enriched in the 4 modules (*P* < 1e-4; Fig. 2B). The results showed that the 6 motifs exhibit a significant positional bias within the range of 200- to 0-bp upstream of the start codon in the promoter regions of genes from the 4 modules and 500 random genes. Furthermore, the frequency and density of the 6 motif occurrences were notably higher in the promoter regions of genes within the selected modules compared to those in the random genes (Fig. 2C). These findings suggested that these 6 motifs were unique to these 4 modules.

To predict the potential TFs that could recognize the motifs, the 6 predicted motifs were compared with the known cis-elements in plant database, including AthaMap, AGRIS, and PLACE (Mahony and Benos 2007; Yilmaz et al. 2011). Several motifs shared high similarities (*E* < 0.05) with the binding sites of TF families including ARF, MYB, and SPL (Supplementary Data Set 2) that have been previously reported as key regulators in modulating N response in Arabidopsis (Gutierrez et al. 2008; Krouk et al. 2010). In addition, we found that Motif 1, the highest enriched motif within the 4 modules, contained a GCC-box (Fig. 2B, Supplementary Data Set 2) which is a core sequence in the binding site recognized by AP2/ERF TFs (Fujimoto et al. 2000). Plant and yeast promoter databases were used to further investigate Motif 1. The results showed that Motif 1 was significantly associated with the N metabolism processes in both databases (Supplementary Data Set 2). Thus, genes containing Motif 1 in their promoter regions may be regulated by an AP2/ERF family member.

### An AP2/ERF TF was identified to interact with Motif 1

To explore the potential AP2/ERF family TFs binding to Motif 1, Y1H assay was performed. To select the sequence matching Motif 1 matrix for Y1H screening, we first chose the genes with more (>60; Supplementary Data Set 2) occurrences of Motif 1 in their promoter regions from the 4 modules; 63 genes were then retained. Considering the position bias and genes more related to N, we then chose the best-matching sequence to Motif 1 from a NRT (GRMZM2G124396, −55 to 20 bp from start codon, 10 occurrences in this region; Supplementary Data Set 2). As a result, a region from −55 to −1 bp of the start codon from this sequence, namely, Motif 1, was chosen for Y1H screening.

The 55-bp sequence was artificially synthesized 4 times and used for the Y1H screening. Among the 19 positive clones, 6 TFs belonging to the AP2/ERF family appeared 14 times during screening, and ZmEREB97 (GRMZM2G068967, which appears as high as 5-fold) was chosen for further analysis (Supplementary Data Set 2). The sequence of ZmEREB97 was located on maize B73_v3 genome chr2, 10794242–10793574, and the full-length protein contained 222 amino acids, with the AP2/ERF domain from residues 18 to 75. The theoretical molecular weight of ZmEREB97 was 22.9 kDa, and the pI was 9.73. To confirm the interaction in the yeast, we retransformed the 4×Motif 1 bait strain with a full-length ZmEREB97–GAL4AD plasmid. As shown in Fig. 2D, ZmEREB97 can interact with the Motif 1 in vitro. Electrophoretic mobility shift assays (EMSAs) were then performed to investigate the binding specificity of ZmEREB97 using a sequence with mutation in the GCC-like sites as the nonlabeled mutated probe. The EMSA result showed that ZmEREB97 can bind to the wild-type (WT) sequence of Motif 1 but not to the nonlabeled mutated sequence (Fig. 2E).

To test whether ZmEREB97 has a transcriptional activator or a repressor activity, we conducted a dual-luciferase transient transcriptional activity assay. The construct 35S::ZmEREB97, with ZmEREB97 ORF, was generated as an effector. The reporter construct contains 2 luciferase cassettes, the Renilla luciferase gene (REN) driven by the cauliflower mosaic virus (CaMV) with the 35S promoter (35S::REN) used as an internal control and the firefly luciferase gene (LUC) driven by the 4×motif that was used as a reporter (Fig. 2F). Coexpression of 4×motif1::LUC with 35S::ZmEREB97 resulted in a 3.1-fold (*t* test, *P* ≤ 0.01) increase in LUC activity compared with the control (Supplementary Fig. S5, Fig. 2F), implying that ZmEREB97 acted as a transcriptional activator.

### Localization and expression of *ZmEREB97* in maize roots

**Figure 3.**
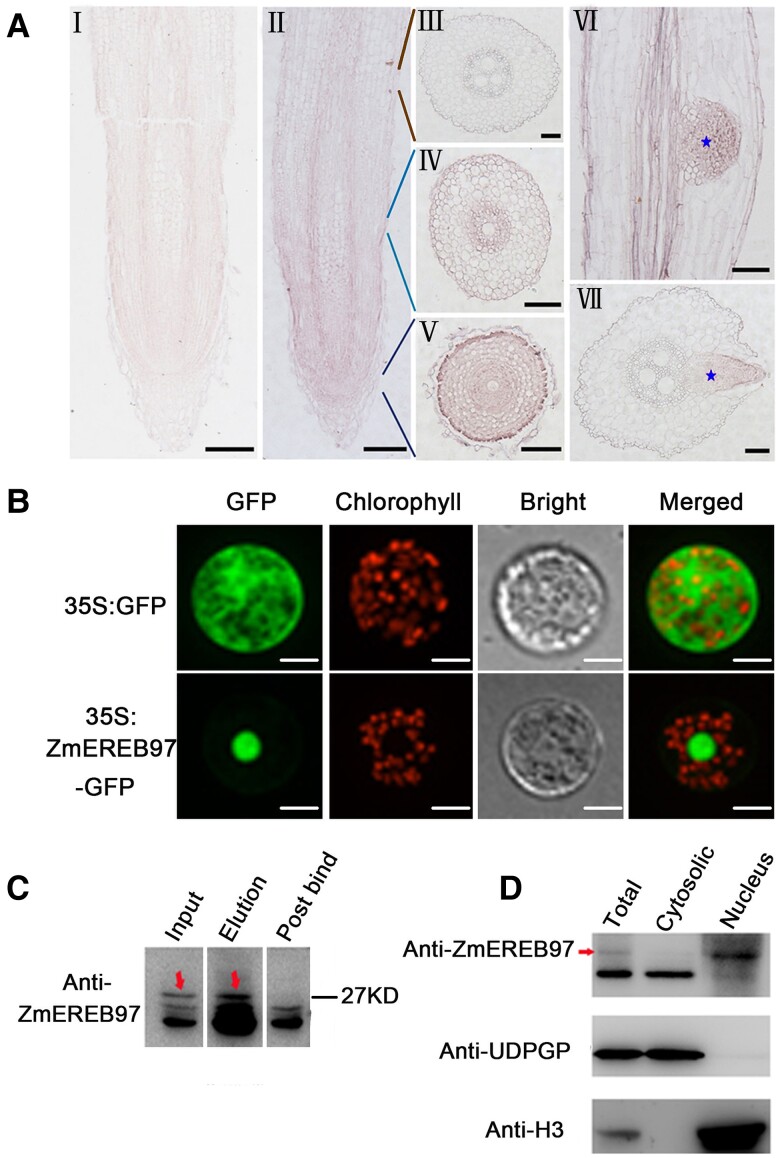
Localization of ZmEREB97 in maize. **A)** RNA in situ hybridization assay to detect transcripts of *ZmEREB97* in maize root. (Ⅰ) Sense probe (negative control). (Ⅱ to Ⅶ) Antisense probe. (Ⅱ) Main root. (Ⅲ) Zone of cell differentiation. (Ⅳ) Zone of cell elongation. (Ⅴ) Zone of cell division. (Ⅵ) Longitudinal section of lateral root. (Ⅶ) Transverse section of lateral root. Hybridization signals detected by labeled antisense probes in lateral root are shown by stars. Bar = 100 *μ*m. **B)** The subcellular localization of ZmEREB97 in maize mesophyll protoplasts. Bar = 20 *μ*m. **C)** Determination of ZmEREB97 antibody specificity in the input sample (input), elution sample (elution), and postbind input sample (post bind) of the immunoprecipitation experiment by western blot analyses. Arrows indicate ZmEREB97-specific band. **D)** Western blot assay of ZmEREB97 in roots of maize seedlings. Total, total proteins; cytosolic, cytosolic proteins; nucleus, nucleus proteins. UDPGP protein was used as a cytosolic marker, and histone H3 was used as a nucleus marker, respectively. Red arrows indicate ZmEREB97-specific band.

To clarify the tissue localization of *ZmEREB97* in maize root, the spatial distribution of its mRNA in the roots was examined by RNA in situ hybridization assay (Fig. 3A). *ZmEREB97* transcripts were mainly expressed in the main root, especially in cell elongation and division zones (Fig. 3A, Ⅱ to Ⅴ), and in the primordial lateral root (Fig. 3A, Ⅵ to Ⅶ).

The biological function of TFs depends highly on their subcellular localization. Therefore, the full-length CDS of *ZmEREB97* driven by CaMV 35S promoter was fused with GFP at the C-terminal. The 35S:ZmEREB97–GFP construct was transiently transformed into maize mesophyll protoplast. As shown in Fig. 3B, the green fluorescence of ZmEREB97–GFP was detected in the nucleus. This localization was confirmed by staining nucleus using the red fluorescent probe and by merging both pictures (Fig. 3B), indicating that *ZmEREB97* was located in the nucleus. Western blot assay of ZmEREB97 in roots of maize seedlings also showed that ZmEREB97 was mainly expressed in the nucleus (Fig. 3, C and D).

**Figure 4.**
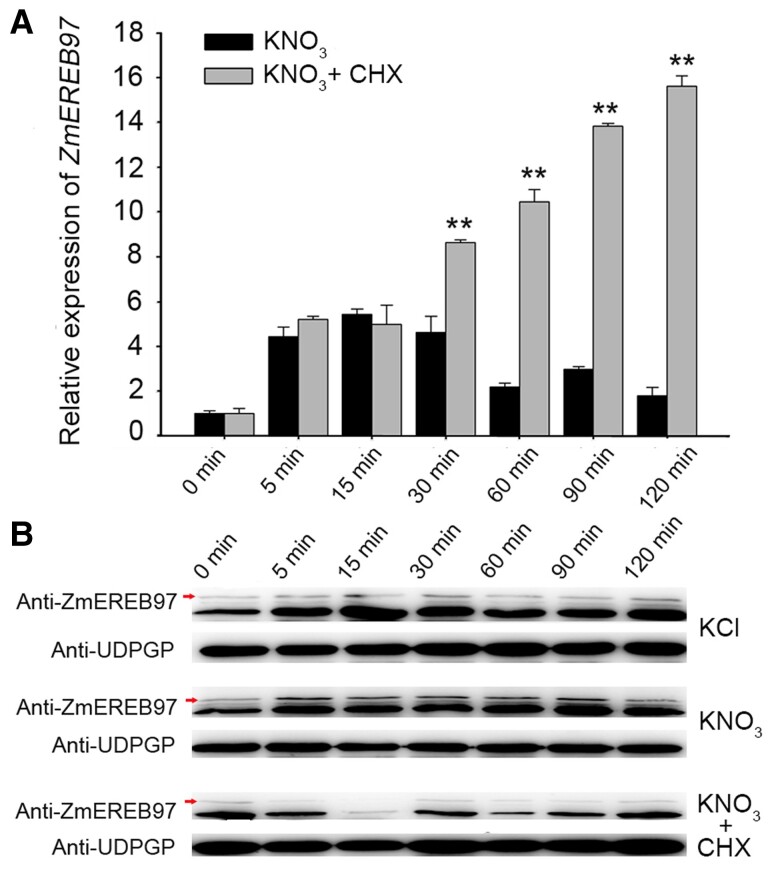
Time course analysis of expression of ZmEREB97 in maize roots with or without CHX under nitrate treatment. **A)** Expression levels were analyzed by qPCR and normalized using *ZmUPF1* as an internal control. Three independent biological replicates were performed with similar results. The bars represent means ± Sd (*n* = 3). ANOVA was used to analyze the expression levels of *ZmEREB97* under different treatments. **P < 0.01. **B)** Western blot analysis of protein levels of ZmEREB97 with or without CHX. Anti-UDPGP was used as a sample loading control, red arrows indicate the ZmEREB97-specific band.

To test whether the expression of *ZmEREB97* is involved in nitrate response, qPCR and western blot analysis were performed. The results of Fig. 4, A and B, showed that mRNA and newly synthesized protein of ZmEREB97 were rapidly accumulated under the nitrate treatment in 5 min and gradually decreased from 60 to 120 min. By contrast, when treating maize roots with cycloheximide (CHX), a chemical reagent that can inhibit protein synthesis (Gilkerson et al. 2016), the accumulation of *ZmEREB97* mRNAs was increased, suggesting that de novo protein synthesis may not be required for *ZmEREB97* expression under nitrate treatment.

### ZmEREB97 binds to the promoter of genes related to N metabolism

**Figure 5.**
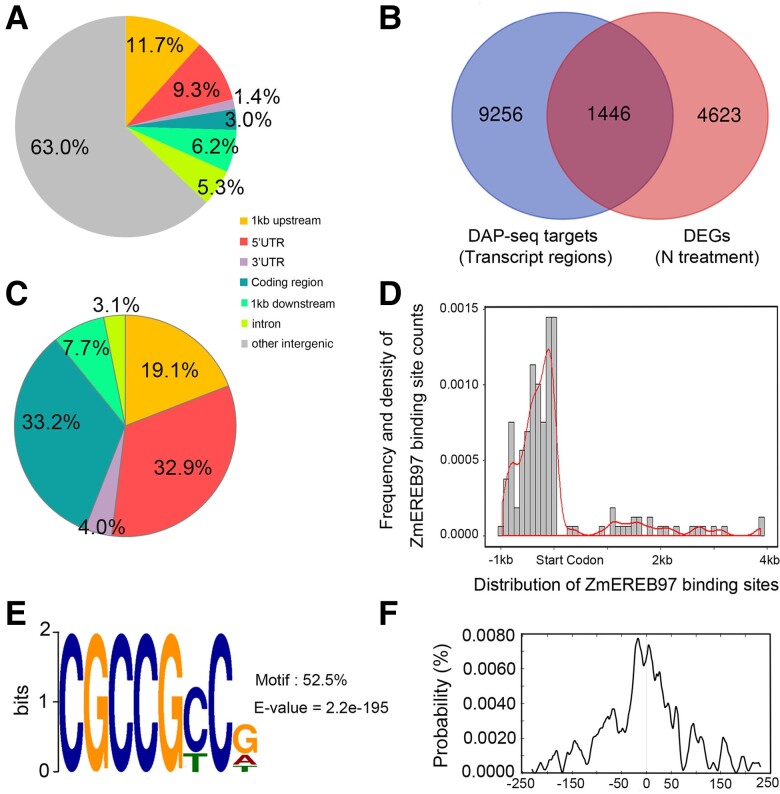
Genome-wide binding profiles from DAP-seq analysis of ZmEREB97. **A)** Distribution of ZmEREB97-binding regions in the maize genome. **B**) Venn diagram showing the overlap of 10,702 genes bound by the ZmEREB97 in the promoter regions to the 6,069 DEGs. Genes in the overlapping regions were identified as potential ZmEREB97-targeted genes. **C)** Distribution of ZmEREB97-binding sites in 1,446 potential ZmEREB97-targeted genes. **D)** Frequency distribution histogram and density map showing the distribution of ZmEREB97-binding peaks in the 1,446 potential ZmEREB97-targeted genes from −1-kb upstream to 4-kb regions. **E and F)** ZmEREB97-binding motifs identified by MEME-ChIP in the 500-bp flanking sequences around the summits of peaks associated with ZmEREB97 potential targets and the density plot of this motif around the summits of the peaks.

To further elucidate genes directly targeted by ZmEREB97, a DAP-seq assay was performed using DNA from inbred maize lines B73 root tissues. ZmEREB97-binding sites were identified from consistent peaks generated by 2 biological replications. With a cutoff *q*-value of 0.05 and the genic regions defined as 1-kb upstream from the transcription start site (TSS) to 1-kb downstream from the transcription termination site (TTS), a total of 11,776 peaks distributed on 10,702 gene regions were identified. Among all the detected peaks, more than half (63.0%) of the peaks were located in the intergenic regions. Of the remaining genic region peaks, most of them were located within either 1-kb upstream regions (11.7% of all peaks) or 5′-untranslated regions (UTRs, 9.3%; Fig. 5A).

By overlapping the 6,069 differentially expressed genes (DEGs) from B73 (Supplementary Fig. S6 and Data Set 3) and the putative targets identified by DAP-seq assay, we found 1,446 DEGs that were directly targeted by ZmEREB97 (Fig. 5B, Supplementary Data Set 3), including 130 genes containing Motif 1 in the promoter regions from the 4 modules (Supplementary Figs. S7 and S8). In total, 2,555 peaks were detected locating in the genic regions of the 1,446 DEGs and of these peaks, of which 840 (32.9%) located in the 5′-UTR and 488 (19.1%) in the 1-kb region upstream from the TSS (Fig. 5C). In agreement with the predicted motif position bias (Fig. 2C), the binding sites are enriched in the vicinity of the start codon sites of nitrate-responsive genes (Fig. 5D).

In addition, MEME-ChIP method was applied to analyze the enriched motifs, using sequences from 250-bp upstream to 250-bp downstream of the genomic regions where 2,555 peak points were located. Consequently, a GCC-box-like element as the top-scoring motif (*E* = 2.2e-195, 52.5% enrichment) was identified (Fig. 5E). By examining the density plot of this motif with the 2,555 peaks, this motif was enriched around the peak point (Fig. 5F).

### The loss of function of ZmEREB97 regulates nitrate uptake and accumulation in maize seedlings

To better characterize the function of *ZmEREB97*, we obtained 2 different kinds of mutants for further analysis. A Mu insertion was found in the promoter of the *ZmEREB97* gene (Supplementary Fig. S9, A and B). A knockout mutant using the CRISPR–Cas9 system in the genetic background of KN5585 was generated (Supplementary Fig. S9, C and D). qPCR revealed that the expression of *ZmEREB97* was significantly reduced in *zmereb97-*_MU_ and *zmereb97-*_CRI_ mutant seedlings (Supplementary Fig. S9E).

We first performed RNA-seq with the WT and *zmereb97-*_MU_ and *zmereb97-*_CRI_ roots in 5-mm KNO_3_ treatment and analyzed 1,446 DEGs targeted by ZmEREB97. Among these 1,446 DEGs, 586 and 603 genes were found to be downregulated in *zmereb97-*_MU_ and *zmereb97-*_CRI_, respectively, as compared with WTs. At the same time, 733 and 708 genes were found to be upregulated. An important observation was made regarding the overlap of downregulated and upregulated genes in both *zmereb97-*_MU_ and *zmereb97-*_CRI_. Specifically, 359 genes were found to be downregulated, while 405 genes were upregulated (Supplementary Fig. S10). This finding suggests that the defect in *ZmEREB97* had a significant impact on nitrate metabolism.

**Figure 6.**
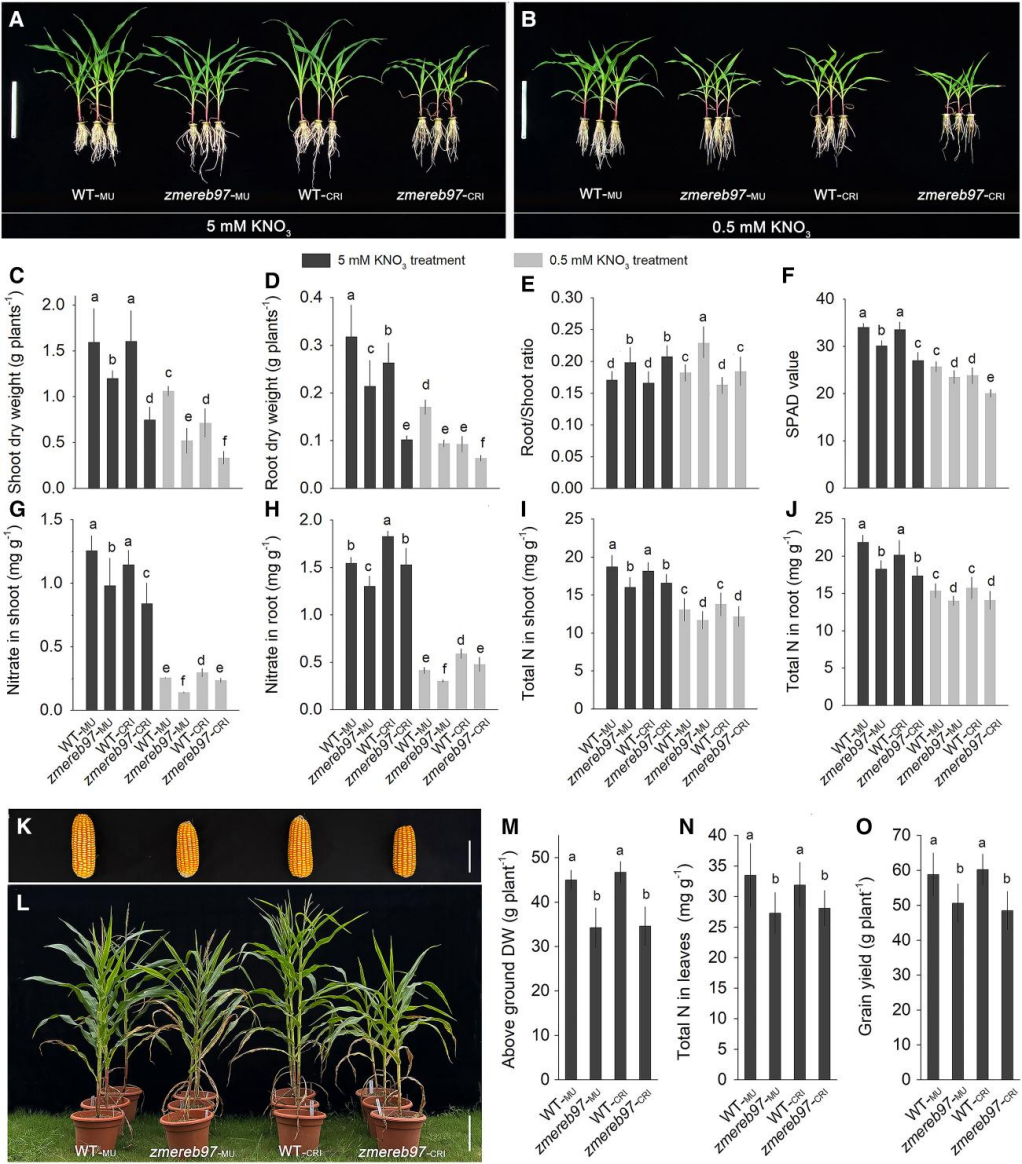
Morphological and physiological phenotypes of *zmereb97* mutants in different nitrate conditions. The plants were grown in 5 mm KNO_3_ for 5 d and then transferred into 5 mm KNO_3_ and 0.5 mm KNO_3_, respectively, for 15 d. **A and B)** The phenotype of WT (WT*-*_MU_ and WT*-*_CRI_) and *zmereb97* mutants (*zmereb97-*_MU_ and *zmereb97-*_CRI_) grown in 5-mm and 0.5-mm KNO_3_ conditions. **C to F)** The biomass, SPAD value, and root/shoot ratio of WT and *zmereb97* mutants under 5-mm and 0.5-mm KNO_3_ treatments. **G to J)** The nitrate and total N content in the root and shoot of WT and *zmereb97* mutants. **K and L)** Phenotype of the ear and aboveground in WT and *zmereb97* mutants on soil condition. **M to O)** The aboveground dry weight, ear length, and grain yield in WT and *zmereb97* mutants in soil-based experiments. Bars = 30 cm in **A)** and **B)**, 6 cm in **K)**, and 35 cm in **L)**. The error bars in **C to J)** and **M to O)** represent means ± Sd (*n* = 6). Lowercase letters indicate significant differences at *P* < 0.01 according to Student's *t* test.

The mutants and WT were then hydroponically cultured under high-NO_3_^−^ (5-mm KNO_3_) and low-NO_3_^−^ (0.5-mm KNO_3_) conditions for 20 d to measure the morphological and physiological phenotypes. As in Fig. 6, A and B, *ZmERER97* mutation caused a notable shoot and root growth inhibition regardless of high or low KNO_3_. Consistently, the shoot and root dry weights of *zmereb97-*_MU_ and *zmereb97-*_CRI_ mutants were significantly lower than those of the WTs (Fig. 6, C and D). The root/shoot ratio exhibited similar responses in WT and mutants under high- or low-KNO_3_ conditions. In the presence of 5-mm KNO_3_ or 0.5-mm KNO_3_ condition, the loss of *ZmEREB97* function all led to an increase in the root/shoot ratio (Fig. 6E). Compared with WTs, the SPAD value of ZmEREB97 mutants was significantly decreased regardless of the 5-mm KNO_3_ or 0.5-mm KNO_3_ condition (Fig. 6F). In addition, we tested NO_3_^−^ content and total N accumulation in these lines. The *zmereb97-*_MU_ and *zmereb97-*_CRI_ mutant plants took up less NO_3_^−^ and showed lower total N accumulation in the roots and shoots as compared with their responsive WT (Fig. 6, G to J). Together, these data confirm that loss of function of ZmEREB97 impairs N uptake and accumulation and subsequently affects plant growth.

To further evaluate the potential contribution of ZmEREB97 to N-dependent yield formation, we planted WT and *zmereb97-*_MU_ and *zmereb97-*_CRI_ mutants under soil condition. As shown in Fig. 6, L and M, the aboveground biomass of *zmereb97-*_MU_ and *zmereb97-*_CRI_ was greatly reduced as compared with the responsive WT. For yield formation, *zmereb97-*_MU_ and *zmereb97-*_CRI_ stably reduced total N accumulation in leaves by ∼15.1% and ∼11.9%, resulting in a decrease in grain yield per plant of ∼13.6% and ∼15.4% compared to responsive WT (Fig. 6, K, N, and O). These findings indicate that loss of function of ZmEREB97 results in reduction in plant growth and grain productivity.

**Figure 7.**
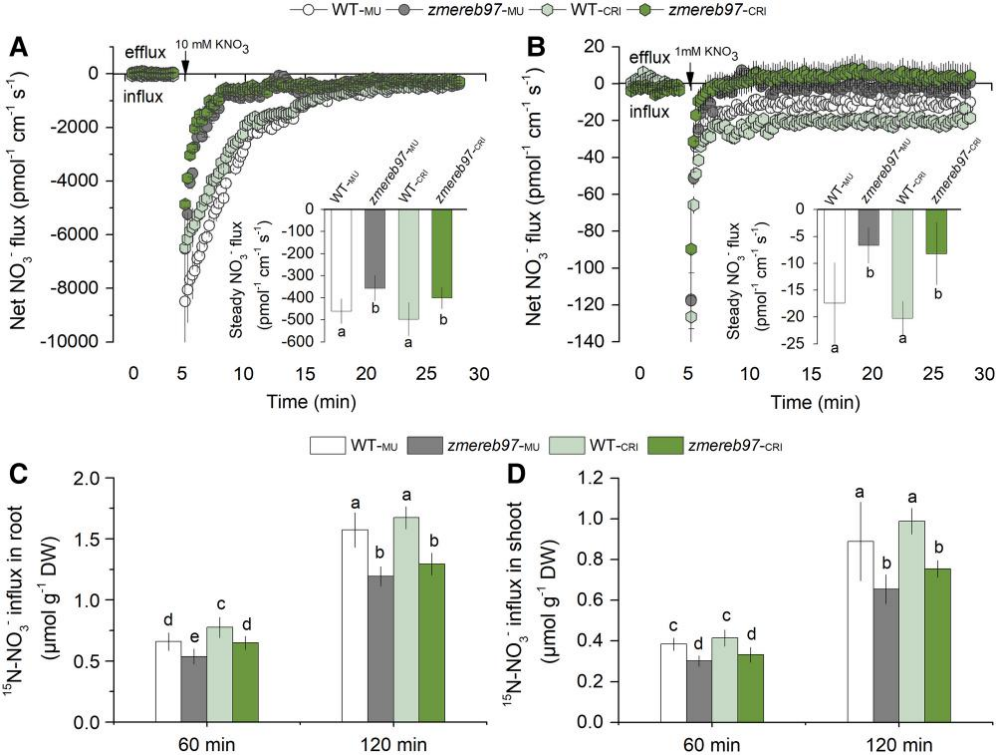
Analysis of net NO_3_^−^ flux, steady NO_3_^−^ flux, and ^15^N-NO_3_^−^ flux in WT and *zmereb97* seedlings. **A and B)** Net NO_3_^−^ flux in root of 7-d-old maize seedlings. Seedlings were transferred into measuring solution with 0.5 mm KNO_3_ for 40 min before measurement. After 5 min of flux recordings, 10 mm KNO_3_ or 1 mm KNO_3_ was added into the measuring solution. Inserts in **A)** and **B)** denote the steady NO_3_^−^ influx after 30-min KNO_3_ treatment. The sign convention is “influx negative.” **C and D)** Maize seedlings of the WT (WT*-*_MU_ and WT*-*_CRI_) and *zmereb97* mutants (*zmereb97-*_MU_ and *zmereb97-*_CRI_) were grown in Hoagland solution for 2 wk and then deprived of N for 4 d. ^15^N-NO_3_^−^ influx of roots and shoots were then measured using 0.5 mm K^15^NO_3_ for 60 and 120 min. The bars represent means ± Sd (*n* = 6). Lowercase letters indicate significant differences at *P* < 0.01 according to Student's *t* test.

We then studied the difference in kinetics of NO_3_^−^ uptake of roots between WT and *zmereb97* mutants. Addition of 10 or 1 mm KNO_3_ to the bath solution resulted in a massive and transient net NO_3_^−^ uptake, with net NO_3_^−^ influx being lower in *zmereb97-*_MU_ and *zmereb97-*_CRI_ root compared with that in WT (Fig. 7, A and B). When roots were incubated in working solution with 10 mm KNO_3_ for 40 min, the substantially lower initial NO_3_^−^ influx was also observed in the root of *zmereb97* mutants (inserts in Fig. 7A); the same trends were observed for 1-mm KNO_3_ treatment (inserts in Fig. 7B). Using a ^15^N-abundance assay, nitrate influx rates in root and shoot of *zmereb97-*_MU_ and *zmereb97-*_CRI_ lines were shown to be lower than in the WT-_MU_ and WT-_CRI_ (Fig. 7, C and D), further indicating that ZmEREB97 may be beneficial for enhancing NO_3_^−^ uptake.

NR is the first enzyme of the N reduction pathway in plants, serving as a bridge connecting nitrate uptake and assimilation. NR is usually considered a marker gene, and its expression and activity can reflect the nitrate level and metabolism in plants (Berger et al. 2020; Cao et al. 2023). Here, we found that the expression of *ZmNR1.1* and *ZmNR1.2* were greatly repressed in *zmereb97-*_MU_ and *zmereb97-*_CRI_ under low- and high-N conditions, respectively (Supplementary Fig. S11, A and B). Consistent with the qPCR results, NR enzyme activity analysis confirmed that the activity of NR was significantly inhibited in *zmereb97-*_MU_ and *zmereb97-*_CRI_ mutants compared with respective WT (Supplementary Fig. S11C). We further assessed the expression of another 2 NO_3_^−^ response marker genes including *ZmGS1* and *ZmGS2* to investigate whether ZmEREB97 is required for NO_3_^−^ signaling. Under 5-mm or 0.5-mm KNO_3_ treatments, the transcriptional induction of *ZmGS1* and *ZmGS2* in roots of both *zmereb97-*_MU_ and *zmereb97-*_CRI_ mutants were largely reduced in comparison with their respective WT (Supplementary Fig. S11, D and E), suggesting that ZmEREB97 is functionally relevant for NO_3_^−^ signaling. In support, we also observed that the loss of function of *ZmEREB97* causes significant reduction in the GS activity (Supplementary Fig. S11F). These results indicate that nitrate reduction could be significantly influenced by the impact of ZmEREB97 on nitrate uptake.

**Figure 8.**
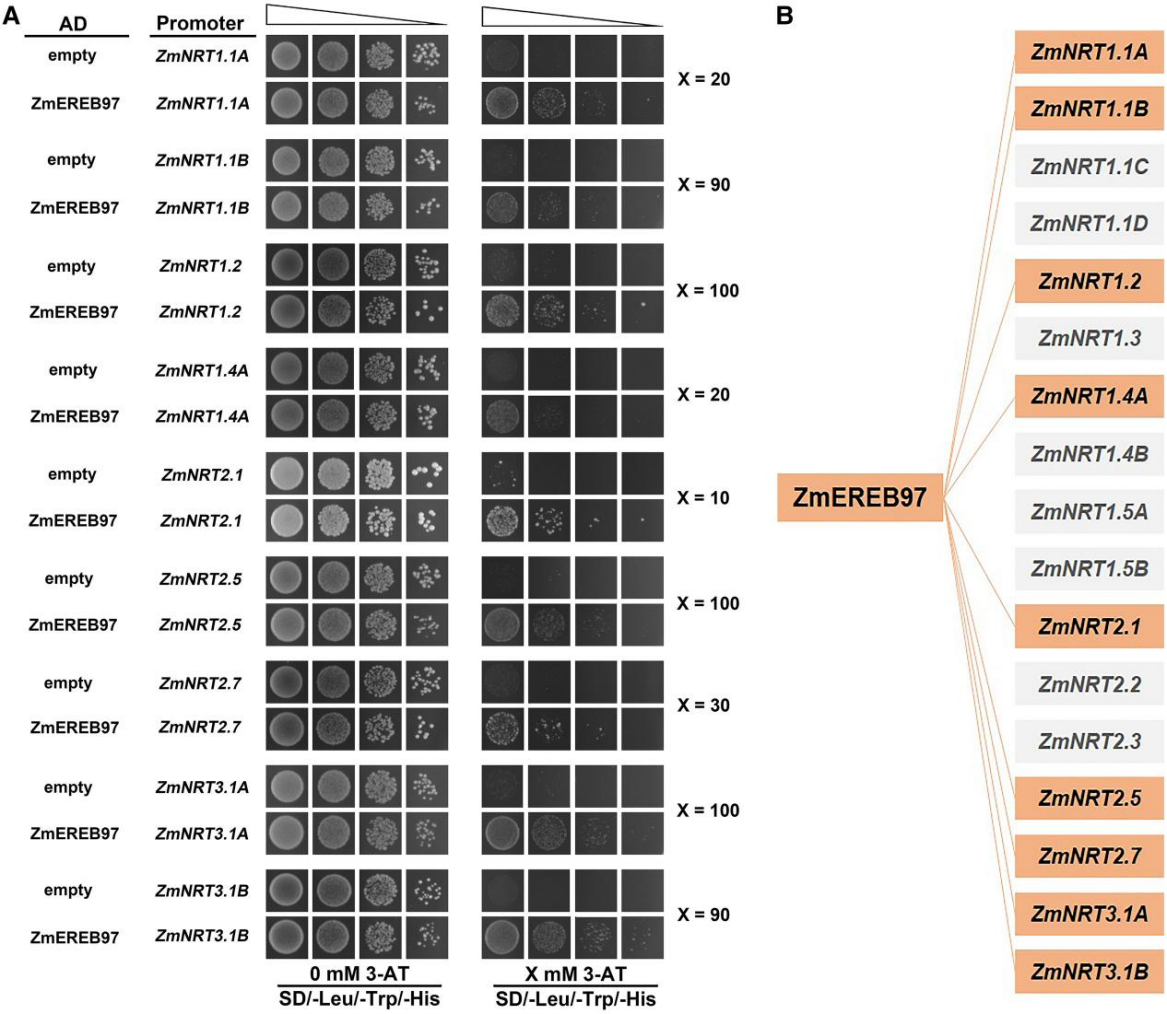
Validation of the interaction of ZmEREB97 on *ZmNRT* gene promoters in maize. **A)** Y1H assay. The interaction was determined on the SD medium lacking Leu, Trp, and His in the presence of different concentrations of 3-amino-1,2,4-triazole (-Leu/-Trp/-His + X mm 3-AT). **B)** Display of potential interaction between ZmEREB97 and 17 *ZmNRT* genes in maize. The genes labeled indicate interaction between ZmEREB97 and 9 *ZmNRT* gene promoters.

To unravel how ZmEREB97 regulates the nitrate uptake in maize, we investigated the link between ZmEREB97 and NRT genes. About 1200 bp upstream region of 17 subgroups of *ZmNRT* genes were fused into the pHIS2.1 vectors, and the bait construct was then successfully generated. The Y1H assay showed ZmEREB97 could interact with the promoter of 9 *NRT* genes including *ZmNRT1.1A*, *ZmNRT1.1B*, *ZmNRT1.2*, *ZmNRT1.4A*, *ZmNRT2.1*, *ZmNRT2.5*, *ZmNRT2.7*, *ZmNRT3.1A*, and *ZmNRT3.1B* (Fig. 8A).

**Figure 9.**
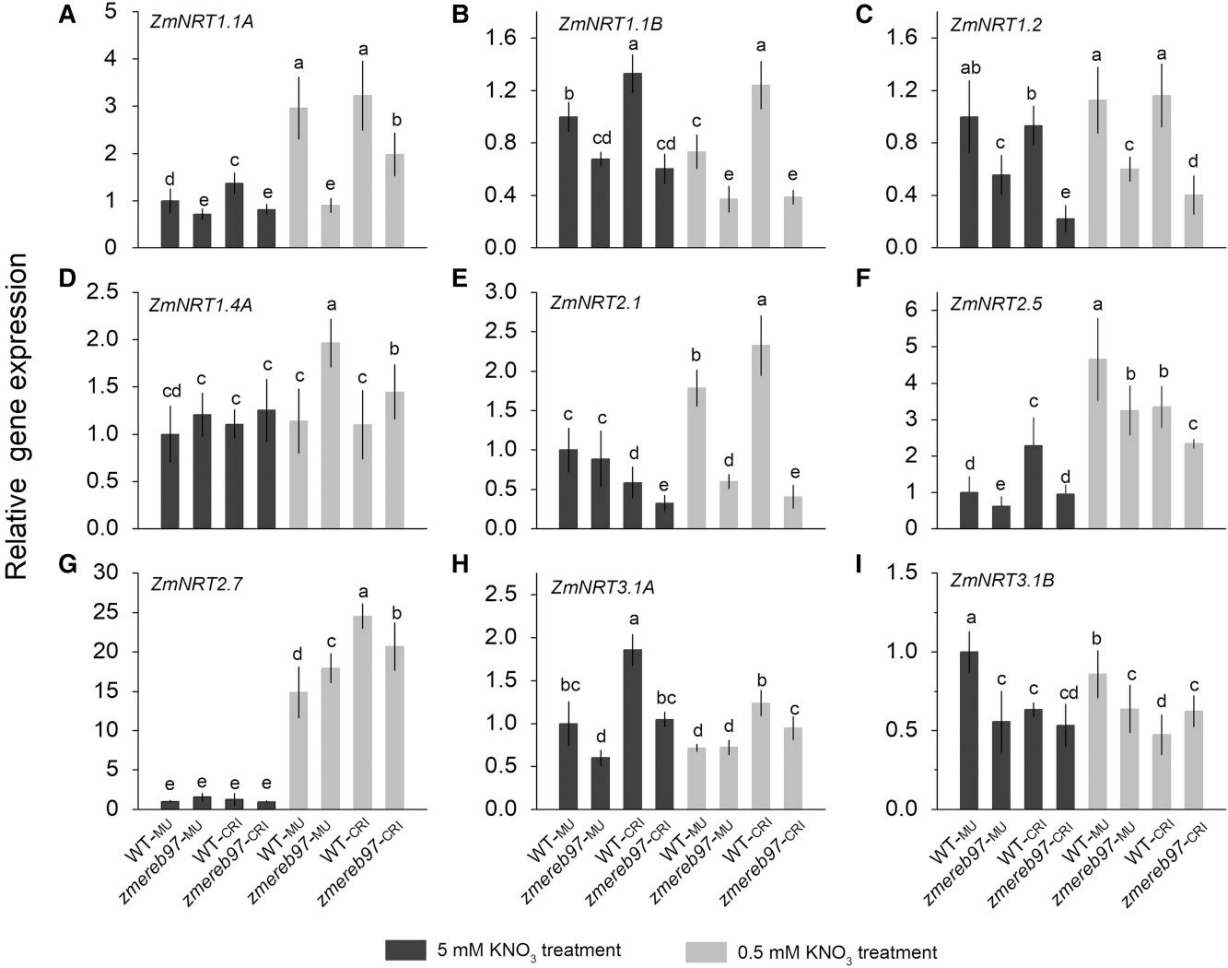
Transcription of transporter genes in the maize root of WT and *zmereb97* mutant seedlings on 5 and 0.5 mm KNO_3_ treatments. Maize seedlings of the WT (WT*-*_MU_ and WT*-*_CRI_) and *zmereb97* mutants (*zmereb97-*_MU_ and *zmereb97-*_CRI_) were grown in Hoagland solution with 5 mm KNO_3_ for 1 wk and then transferred to 5 and 0.5 mm KNO_3_ treatments for 3 d. All transcript levels were quantified by RT-qPCR and normalized to housekeeping genes (*ZmUPF1*). The bars represent means ± Sd (*n* = 3). Lowercase letters indicate significant differences at *P* < 0.01 according to Student's *t* test (*n* = 3 collective samples each containing 3 biological replicates).

To verify the interaction determined by Y1H, we performed transcript-level analyses in maize roots supplied with 5 or 0.5 mm KNO_3_. Compared with the WT, *ZmNRT1.1A* transcripts were significantly decreased in the *zmereb97-*_MU_ and *zmereb97-*_CRI_ mutants regardless of nitrate concentration (Fig. 9A). Decreases in transcript levels were also found for 2 additional *ZmNRT1* genes (*ZmNRT1.1B, ZmNRT1.2*) in the mutants (Fig. 9, B and C). Among the 3 genes belonging to the *ZmNRT2* family, the expressions of *ZmNRT2.1* and *ZmNRT2.5* in roots were significantly inhibited in the *zmereb97* mutants under the 0.5-mm KNO_3_ condition, whereas the *ZmNRT2.5* was not obviously affected (Fig. 9, E to G). Besides, we observed decreased expression of *ZmNRT3.1A* in *zmereb97* mutants in comparison with WT (Fig. 9H). Collectively, 6 genes of *ZmNRT1.1A*, *ZmNRT1.1B, ZmNRT1.2*, *ZmNRT2.1*, *ZmNRT2.5*, and *ZmNRT3.1A* were shown to be strongly affected when the function of *ZmERER97* was lost.

**Figure 10.**
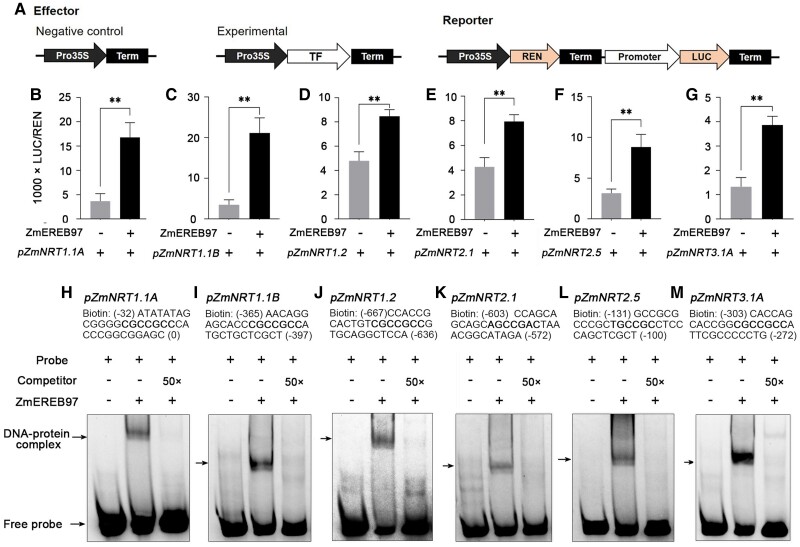
The transcriptional regulation of ZmEREB97 on 6 *ZmNRT* genes. **A)** Schematic diagrams of the effector and reporter constructs. “REN” represents Renilla luciferase gene; “LUC” firefly luciferase gene; “Term” terminator; “Pro35S” the promoter of CaMV 35S; “TF” the cDNA of ZmEREB97; and “Promoter” the promoter of *NRT* gene. **B to G)** The promoter activities of *ZmNRT1.1A*, *ZmNRT1.1B*, *ZmNRT1.2*, *ZmNRT2.1*, *ZmNRT2.5*, and *ZmNRT3.1A* activated by ZmEREB97 LUC assays were expressed as the ratio of LUC to REN in *N. benthamiana* leaves cotransformed with the effector and the reporter combinations. The bars represent means ± Sd (*n* = 3). Two-tailed Student's *t* test (*n* = 3 collective samples each containing 3 biological replicates) was used to test the significance. **P < 0.01. **H to M)** EMSA using probes derived from the ZmEREB97 target gene promoters containing GCC-box-binding sites. + and − indicate the presence and absence of the indicated probe or protein, respectively.

To further confirm the above interaction, a transient transcription dual-luciferase assay was performed in *Nicotiana benthamiana* leaves, using ZmEREB97 driven by the CaMV 35S promoter as an effector and LUC (the coding region of firefly luciferase) driven by the different gene promoters as reporters (Fig. 10A). As shown in Fig. 10, B to G, coexpression of *NRT* gene promoters::LUC with 35S::ZmEREB97 resulted in a significant increase in the LUC activity compared with the 35S::ZmEREB97 alone. As for *ZmNRT1.1A*, *ZmNRT1.1B*, *ZmNRT1.2*, *ZmNRT2.1*, *ZmNRT2.5*, and *ZmNRT3.1A* gene promoters, when ZmEREB97 was coexpressed with them, the LUC/REN values increased by 3.1-, 4.8-, 0.7-, 1.7-, 0.9-, and 1.9-fold, respectively.

The family of AP2/ERF TFs has been shown to recognize and bind to GCC-box element (Shoji and Yuan 2021). Since the GCC-box-like element was identified as the top-scoring motif in our experiments (Fig. 5E), it prompted us to examine whether ZmEREB97 binds to the GCC-box in the promoters of *NRT* genes. The EMSA showed ZmERER97 could bind in vitro to probes containing GCC-box in each promoter of these genes, and the binding activity was partially abolished by the addition of 50-fold cold competitor probes (Fig. 10, H to M).

## Discussion

TFs control diverse biological processes by binding to the promoters of target genes, and a number of TFs have been identified that control N assimilation (Vidal et al. 2015). For example, the overexpression of a member of the AP2/ERF, *OsDREB1C*, not only boosts grain yields but also confers high NUE in rice (Wei et al. 2022). Maize is an essential dual-use food and energy crop predominantly cultivated in aerobic soils where nitrate is often the primary source of N available for growth (Wen et al. 2017). A better understanding of the regulatory system of maize to maximize nitrate uptake and utilization is therefore a critical step toward deciphering the molecular mechanism of nitrate use in maize. Here, we also identified an AP2/ERF TF, ZmEREB97, and reported its functional characterization in modulating the nitrate response and uptake, suggesting that it was a potential candidate for improving NUE in maize.

### ZmEREB97 is a unique AP2/ERF gene modulating nitrate uptake

The AP2/ERF superfamily is one of the largest groups of TFs in plants and plays important roles in the abiotic stress response and developmental processes (Licausi et al. 2013). Up to now, this family was also mainly implicated in maize developmental processes and response to stress conditions (Zhuang et al. 2010). Here, we show that a TF from AP2/ERF family, ZmEREB97, plays a critical role in mediating plant responses to nitrate availability. Several lines of evidence support this claim. First, the expression of ZmEREB97 can be rapidly induced by nitrate in root (Fig. 4), confirming its potential role in the nitrate response. Second, RNA in situ hybridization assay indicated that *ZmEREB97* was preferentially expressed in the main root, particularly in cell elongation and division zones, as well as in the primordial lateral root (Fig. 3A), suggesting that ZmEREB97 may exert function in nitrate uptake. Third, DAP-seq assay showed that ZmEREB97 could bind to 130 genes containing Motif 1 in the promoter regions from the 4 N response modules (Fig. 2, D and E, Supplementary Fig. S8). Motif 1, which contains the GCC element, has been identified as the most enriched motif among the 4 N response modules (Fig. 2, A to C). These data strongly suggest that ZmEREB97 is an essential regulator in nitrate-responsive network in maize.

As N is a key factor limiting plant growth and crop productivity, the loss of function of N regulatory TFs is often accompanied by plant phenotype changes. Some established regulators of N status such as OsDREB1C, OsTCP19, AtNLP7, AtTCP20, ZmNLP5, and ZmM28 were shown to exert limitation to plant growth (Marchive et al. 2013; Guan et al. 2017; Wen et al. 2017; Wu et al. 2019; Ge et al. 2020; Liu et al. 2021). Consistent with previous reports, *zmereb97* mutants exhibited a weakened phenotype under both sufficient and limiting nitrate conditions (Fig. 6, A, B, and L), highlighting the crucial role of ZmEREB97 as a key regulator of N response network. In the course of this work, *ZmEREB97* mutation in maize plants resulted in substantial shoot biomass reductions under both hydroponic (Fig. 6C) and soil conditions (Fig. 6M), and these biomass decreases were accompanied by a less grain yield (Fig. 6O). Plants respond to N availability by changing their root to shoot ratios. One hypothesis used to explain this allocation is that plants optimize their behavior by maximizing their relative growth rate (Agren and Franklin 2003). The *zmereb97* mutants displayed higher root to shoot ratio under sufficient and limiting nitrate conditions (Fig. 6E). Given the significant biomass reduction caused by *ZmEREB97* mutation, we propose that ZmEREB97 may act as a positive regulator in influencing the efficiency of nitrate acquisition from maize root.

We next present physiological evidence in support of the assumption that ZmEREB97 confers promoted nitrate acquisition. Analysis of nitrate and total N content in both roots and shoots revealed a significant decrease in NO_3_^−^ and total N accumulation in 2 *zmereb97* mutant lines compared to WT plants (Fig. 6, G to J and N). The lower nitrate and N accumulation in mutation lines may be attributed to the inhibited influx of NO_3_^−^ in root tissues. The following results demonstrated that the net NO_3_^−^ influx and steady NO_3_^−^ influx in mature root surface were remarkably reduced in *zmereb97* mutants (Fig. 7, A and B). Likewise, the lower ^15^NO_3_^−^ influx was also observed in root and shoot of *zmerer97*-_MU_ and *zmerer97*-_CRI_ lines (Fig. 7, C and D). Here, we established that the function of ZmEREB97, which operates maize growth, is significantly associated with its ability to regulate nitrate uptake and accumulation.

### ZmEREB97 is involved in nitrate transport through regulating *ZmNRT* gene expression

Changes in nitrate assimilation are accompanied by changes in transcription (Gaudinier et al. 2018). Given that the ZmEREB97 has a positive effect on nitrate uptake, it is expected that it may have multiple downstream targets and distinct translational regulation. NRTs play a crucial and extensive role in taking up nitrate from the soil and transporting it to different organs for plant growth and development (Glass 2003). In maize, a total of 78 *NRT1*, 7 *NRT2*, and 2 *NRT3* genes were identified (Jia et al. 2023). In this study, 17 subgroups of key *ZmNRT* genes including 10 Zm*NRT1*, 5 Zm*NRT2*, and 2 Zm*NRT3* genes were selected to verify their potential interaction with ZmEREB97. Based on the results of Y1H assay, 9 *ZmNRT* genes associated with nitrate transport were identified to interact with ZmEREB97 (Fig. 8). qPCR analysis of these 9 genes demonstrated that the expression of 6 genes of *ZmNRT1.1A*, *ZmNRT1.1B*, *ZmNRT1.2*, *ZmNRT2.1*, *ZmNRT2.5*, and *ZmNRT3.1A* were significantly downregulated in *zmereb97* mutants as compared with WT (Fig. 9). Within the *ZmNRT* gene family, the function and nitrate expression patterns were different. *ZmNRT1.1A* has been shown to enhance expression in shoots and improve growth under N deficiency stress (Sakuraba et al. 2021). Very recently, overexpressing *ZmNRT1.1B* was proven to determine N use efficiency, and its overexpression confers significantly higher grain yield under low to moderate N supply in the fields (Cao et al. 2023). *ZmNRT2.1* has improved functional interaction in nitrate uptake along the root axis of maize, as evidenced by gene expression results (Lupini et al. 2016). The expression of *ZmNRT2.5* was upregulated with prolonged N starvation and reduced after nitrate resupply, indicating it may play an important role in mediating nitrate uptake by plants under long-term low-N stress. On the contrary, *ZmNRT3.1A* displayed decreased expression levels during prolonged N starvation but exhibited a rapid increase in expression after nitrate resupply (Jia et al. 2023). Because all 6 genes targeted by ZmEREB97 are closely associated with different nitrate translocations, it is reasonable to speculate that ZmEREB97 serves as a hub and a positive regulator in mediating NRTs.

AP2/ERF TF family members contain motifs and domains involved in transcription activation, repression, or protein–protein interactions, indicating diverse roles in gene regulation (Chandler 2018). However, much less is known about its function in regulating *ZmNRT* genes in maize. Luciferase-based transient transactivation assays verified that ZmEREB97 activates the expression of 6 interacted *ZmNRT* genes involved in nitrate transport (Fig. 10, B to G). The activating role of ZmEREB97 is also in accordance with the decreased expression levels of these *ZmNRT* genes in the *zmereb97* mutants in our study (Fig. 8). These results suggest that ZmEREB97 functioned as a transcription activator by binding to the promoters of *ZmNRT* genes.

TFs induce or repress gene expression by binding to specific motifs present in the promoters of target genes (Yamasaki et al. 2012). Within the AP2/ERF TF family, DREB subfamily can bind to a consensus A/TGCCGCC sequence and GCC-box-related sequences in the promoter of many target genes (Mizoi et al. 2012). The sequence analysis of the promoters of 6 *ZmNRT* target genes shows that all contain the GCC-box-related sequences. EMSA confirmed that GCC-box elements are necessary for ZmERER97 binding (Fig. 10, H to M). This finding is similar to the previous studies that 2 AP2/ERF TFs, ERF1B and ERF104, can bind to the GCC-boxes in the *NRT1.8* promoter region, thus upregulating *NRT1.8* in Arabidopsis (Zhang et al. 2014). Recently, another AP2/ERF TF in rice, OsDREB1C, was found to bind to the promoters of *OsNRT1.1B* and *OsNRT2.4* and activate their expression in roots (Wei et al. 2022), indicating that the regulation of *NRT* by AP2/ERF TFs may be common in plants.

In conclusion, we demonstrate that the TF ZmEREB97 may serve as a positive regulator of *ZmNRT* gene expression, thereby conferring NUE through the activation of nitrate uptake in maize. The discovery of the role of ZmEREB97 in regulating nitrate response advances our understanding of the regulatory networks and a mechanistic basis of plant responses to N availability. Manipulating *ZmEREB97* expression may be a promising strategy for future biotechnologies to breed high NUE maize to adapt to fluctuating N supply.

## Materials and methods

### Plant materials and growth conditions

Maize (*Z. mays* L.) *zmereb97*-_MU_ mutant in B73 background was obtained from a Mu insertional library (ChinaMu, http://chinamu.jaas.ac.cn/; Liang et al. 2019). Homozygous mutant plants were identified by PCR using the specific primers listed in Supplementary Table S1. The mutant were backcrossed to the B73 genetic background for 3 generations, and then, the WT and positive plants from 1 maize cob were self-pollinated for 2 generations.

Maize *ZmEREB97* CRISPR–Cas9 transgenic lines named *zmereb97*-_CRI_ (in the KN5585 background) were generated by the WIMI Biotechnology Company. The 170-bp guide RNA target editing sequence was selected. Eleven CRISPR/Cas9-edited plants were crossed with KN5585 inbred lines to obtain construct-free materials. The mutants were backcrossed to the KN5585 genetic background for 2 generations, and then, the heterozygote plants were self-pollinated for 1 generation. The WT-_CRI_ and *zmereb97*-_CRI_ mutant was identified and obtained from the same corn cob of the self-pollinated plants.

For hydroponic experiments, after 3 d of germination on wet filter paper, maize seedlings were transferred and grown in modified half-strength Hoagland liquid medium containing 5 mm CaCl_2_, 2 mm MgSO_4_, 0.05 mm EDTA–Fe–Na salt, 0.5 mm KH_2_PO_4_, 50 *μ*m H_3_BO_4_, 10 *μ*m MnCl_2_, 1 *μ*m ZnSO_4_, 0.3 *μ*m CuSO_4_, and 0.5 *μ*m Na_2_MoO_4_. The pH was adjusted to 5.8. The concentration of KNO_3_ was determined based on the experimental requirement. Normally, the seedlings of WT and *ZmEREB97* mutants were grown in Hoagland solution with 5 mm KNO_3_ for 1 wk and then transferred to 5-mm KNO_3_ (HN) and 0.5-mm KNO_3_ (LN) Hoagland liquid solution for 2 wk.

For soil-based experiments, the soil collected from the field was thoroughly mixed with a cement mixer and then evenly divided into uniformly sized cultivation pots. The soil used possessed 1.66 g N kg^−1^ of extracted mineral N, together with 23.1 mg kg^−1^ of available phosphorus (P), 129.6 mg kg^−1^ of available potassium (K), and 32.4 g kg^−1^ of organic matter. To ensure sufficient N and other nutrient elements in the soil, the maize seedlings were watered with Hoagland solution containing 5 mm KNO_3_ 3 times during the entire growth period.

### Transcriptome analysis

Total RNA from 36 samples per treatment was used for transcriptome analysis. Library construction and sequencing were performed according to Illumina instructions by the Berry Genomics Company (Beijing, China), after quality control process. Top Hat was used to align the reads to the maize B73_v3 genome (Trapnell et al. 2009). Fragments per kilobase of exon per million fragments mapped (FPKM) were calculated, representing the expression level. DEGs between control and nitrate-treated samples were calculated. The predicted gene function in this study is based on the annotation provided in MaizeGDB.

### De novo motif discovery and analysis

The MEME Suit (http://meme-suite.org/) was used to identify motifs in the promoter regions of genes from the N-related modules. We defined the promoter regions as 1 kb upstream and 100-bp downstream of the start codon sites. For each module, 50 motifs were generated by MEME with the length from 10 to 25 bp, and the motif with *E* < 0.05 was retained. MAST was used to identify the motif similarities (*P* < 1e-4), and motifs with the similarities >0.6 were merged by STAMP. FIMO was used to identify the enrichment (occurrences) of the motifs in the promoter regions of genes (*P* < 1e-4).

### Y1H analysis

For Y1H screening, 4×Motif 1–HIS was constructed by inserting of sequence (5′-CGACCTCCTCGGTCCACGCCGCCCGTCGGGGTTCGCCAGCCTCCTGGCCGCGCTC-3′) in the multiple cloning sites of pHIS2.1 (Rose and Broach 1991). The cDNA was linked with linearized pGAD T7–DEST vector and used to cotransform with the reporter plasmids into yeast strain Y187. Transformants were grown on SD/-Leu/-His/-Trp plates containing 60 mm 3-aminotriazole (3-AT) for 3 to 5 d at 30 ℃ for selection.

For Y1H assay, the promoter region (−1 bp to approximately −1,200 bp) of *NRT* gene was fused into the pHIS2.1 to generate the bait construct and then transformed into Y187 yeast strain. The coding region of ZmEREB97 was linked with linearized pGADT7–DEST vector as a prey construct. The transformed yeast cells were grown at 30 °C for 4 d on Synthetic Drop-out (SD) medium plates lacking leucine (Leu), tryptophan (Trp), and histidine (His) in the presence of different concentrations of 3-amino-1,2,4-triazole. The prey fragments from the positive colonies were identified by DNA sequencing.

### EMSA

Full-length *ZmEREB97* cDNA was subcloned into pCold (Takara). His-tagged recombinant protein was expressed in *E. coli* host strain DE3 cells. The culture solution was added with IPTG (final concentration 0.4 mm) and kept on culturing at 15 ℃, for 24 h. His-tagged recombinant protein was purified using His-tag Protein Purification Kit (catalog no. P2226, Beyotime, China). Oligonucleotide probes were synthesized and labeled with biotin at the 5′-end. About 80 ng of purified proteins was mixed with the 2.5 ng of probes at 25 ℃, for 20 min in an EMSA/Gel Shift Binding Buffer (Beyotime). The DNA in the gel was then transferred to N^+^ nylon membranes (0.2 *μ*m, Millipore). The DNA on the membrane was detected using Chemiluminescent EMSA Kit (Beyotime).

### Transient transcriptional activity assay

For the transient transcriptional activity assays of the promoters, the 4×motif1 were cloned into vector pGreen II-0800-LUC to generate reporters for the dual-luciferase assays. The full-length ZmEREB97 cDNA was inserted into vector pMDC83-35S to generate a 35S promoter–driven ZmEREB97 effector. The plasmids carrying effectors and reporters were transformed into and *Agrobacterium tumefaciens* GV3101 (pSoup-P19), respectively. Reporter and reference values were measured using Dual-Luciferase Reporter Gene Assay Kit (Beyotime) and BioTek Cytation 5 (BioTek) Microplate Reader. Promoter activity was calculated as the firefly luciferase (fLUC)/Renilla luciferase (rLUC) ratio.

### qPCR analysis

Total RNA was extracted using the SV Total RNA Isolation System Kit (Promega, USA). Prime Script RT Reagent Kit (Takara) was used to reverse-transcribe RNA to cDNA. qPCR was performed in 96-well plates using a Bio-Rad CFX96 system with SYBR Premix Ex Taq II (Takara). The housekeeping genes *ZmUPF1* and *ZmDUF* were used as internal control genes. Three independent biological replicates were used for qPCR analysis.

### Western blot assay

For western blot analyses, total proteins were extracted from B73 roots. Nucleus proteins and cytosolic proteins were isolated by CelLytic PN Isolation/Extraction Kit (Sigma). The protein concentration was quantified by BCA Protein Assay Kit (Beyotime) and resolved on SDS–PAGE. The resolved protein was transferred to the PVDF membrane (0.2 *μ*m, Beyotime), and the membrane with protein samples attached was incubated with 5% nonfat milk, primary antibodies, and secondary antibodies. The antibody against ZmEREB97 was used at 1:1,000; the antibody against UDPGP and H3 was used at 1:3,000.

### DAP-seq

For DAP-seq, the genomic DNA was extracted from 2-wk-old maize inbred line B73 roots, and the genomic library was constructed according to the protocol of Bartlett (Bartlett et al. 2017). Full-length *ZmEREB97* cDNA was subcloned into pFN19K expression vector, which contains an N-terminal HaloTag affinity tag and then expressed in an in vitro wheat germ system (Los et al. 2008). The Halo-ZmEREB97 was immobilized on Magen HaloTag beads, washed, and then incubated with the genome DNA library. DNA was eluted and amplified with indexed TruSeq primers after bead washing. Sequencing was performed on an Illumina HiSeq 2500 with 100 bp SR reads. Input DNA and GFP were used as control, and 2 technical replicates were used for each sample (Ning et al. 2019). Reads were mapped to the maize B73_v3 genome, and DAP-seq peaks were called by MASC2 (Zhang et al. 2008). Peaks were identified with the cutoff *q*-value of 0.05. MEME-ChIP was used for de novo motif discovery (Machanick and Bailey 2011).

### Subcellular localization of *ZmEREB97*

A full-length *ZmEREB97* cDNA, without termination codon, was cloned into vector pAN580 and fused to the N-terminal of GFP. The fusion construct pAN580–ZmEREB97–GFP and the control pAN580–GFP were transformed into maize mesophyll protoplasts independently. Maize mesophyll protoplast isolation and transformation methods were performed according to the protocol described by Tu et al. (2020). The fluorescence signals were captured by a confocal laser scanning microscope (UltraView VOX, PerkinElmer). The settings used for confocal microscopy were as follows (in nanometers: excitation [ex] and emission [em]): for sGFP, ex 488, em 500 to 550; for RFP, ex 552, em 575 to 625; fluorescence intensity, 5%; and gains value, 500 to 800.

### RNA in situ hybridization

A 291 bp specific fragment of *ZmEREB97* was amplified by PCR with the primers (Supplementary Table S1) and inserted into pClone007 Blunt Vector (TsingKe Biotech) for sequencing. Sense probe was generated by primers T7-F and *ZmEREB97*-R and the antisense probe by primers *ZmEREB97*-F and T7-R. Sense and antisense probes were transcribed in vitro from the T7 promoter with T7 RNA polymerases using the digoxigenin RNA Labeling Kit (Roche). Two-week-old maize inbred line B73 root tissues were fixed overnight in 4% (w/v) paraformaldehyde in phosphate buffer, pH 7.0, and embedded in Paraplast Plus (Sigma-Aldrich) for in situ hybridization. As described by Coen et al. (1990), nonradioactive RNA in situ hybridization with digoxigenin-labeled sense and antisense probes were performed on 8-mm sections of different root parts.

### Measurement of net NO_3_^−^ fluxes and nitrate content

Net NO_3_^−^ fluxes were measured noninvasively with NO_3_^−^-selective microelectrodes using the NMT system (BIO-003A system; Younger USA Science and Technology). The working principle and measurement procedure were described in detail by Tang et al. (2012). Maize seedlings were grown in half-strength Hoagland nutrient solution for 2 wk and then transferred to half-strength Hoagland containing 1 mm KNO_3_ for 1 d. Prior to the flux measurements, the ion-selective electrodes were calibrated using NO_3_^−^ concentrations of 0.05 and 0.5 mm. During the entire measurement process, the shoot was not in contact with the measuring solution. The net fluxes of NO_3_^−^ at the meristem were measured individually. Each plant was measured once. The final flux values were the means of more than 5 individual plants. The measuring solution was composed of 0.2 mm CaCl_2_, 0.1 mm NaCl, 0.1 mm MgSO_4_, 1 mm KNO_3_, and 0.3 mm MES (pH 6.0, adjusted with 1 M NaOH).

The influx rate of ^15^NO_3_^−^ was assayed as already described (Tang et al. 2012) on plants grown hydroponically. Maize seedlings were grown in Hoagland solution for 2 wk and then deprived of N for 4 d. The plants were transferred first to 0.1 mm CaSO_4_ for 1 min, then to a complete nutrient solution containing 0.5 mm K^15^NO_3_ for 10 and 30 min, and finally to 0.1 mm CaSO4 for 1 min. After grinding in liquid N, an aliquot of the powder was dried to a constant weight at 70 °C. About 10 mg of powder of each sample was analyzed using the isotope ratio mass spectrometer system (Elementar IsoPrime100). Influx of ^15^NO_3_^−^ was calculated from the ^15^N concentrations of the roots and shoots.

For plant nitrate measurement, maize seedlings of the WT and *zmereb97* mutant were grown in half-strength Hoagland solution for 2 wk and then N starvation for 5 d before measurement. Nitrate concentration was determined as described previously (Leleu and Vuylsteker 2004). Total N concentration in plants was determined by the Kjeldahl method (Li et al. 2006).

### Statistical analysis

All experiments were carried out with at least 3 independent biological replicates. Each measurement was carried out in triplicate. Data represent the mean ± Sd of 3 biological replicates and each containing 5 plant samples. Data were statistically analyzed by ANOVA performed using SPSS Statistics 20.

### Accession numbers

Sequence data from this article can be found in the GenBank database or the MaizeGDB database under the following accession numbers: *ZmEREB97*, Zm00001d002364; *ZmNRT1.1A*, Zm00001d024587; *ZmNRT1.1B*, Zm00001d029932; *ZmNRT1.2*, Zm00001d036941; *ZmNRT1.4A*, Zm00001d044529; *ZmNRT2.1*, Zm00001d054057; *ZmNRT2.5*, Zm00001d011679; *ZmNRT2.7*, Zm00001d044504; *ZmNRT3.1A*, Zm00001d017095; *ZmNRT3.1B*, Zm00001d003287; *ZmNR1.1*, Zm00001d018206; *ZmNR1.2*, Zm00001d052139; *ZmGS1*, Zm00001d026501; and *ZmGS2*, Zm00001d033747.

## Supplementary Material

kiae277_Supplementary_Data

## Supplementary data

The following materials are available in the online version of this article.

**Supplementary Figure S1.** Phenotypic and physiological analysis of B73 and Mo17 seedlings from deficient N condition to sufficient N condition.

**Supplementary Figure S2.** Metabolism overview showing transcriptional profile during KCl and KNO_3_ treatment at 0 and 8 h in B73 and Mo17 root tissues.

**Supplementary Figure S3.** Correlation scatter plots of GS and MM in different modules.

**Supplementary Figure S4.** Correspondence of B73 set-specific and B73-Mo17 consensus modules.

**Supplementary Figure S5.** Transcription activity assay of ZmEREB97 in *Nicotiana benthamiana* leaves.

**Supplementary Figure S6.** Summary plot of consensus eigengene networks and their differential analysis in 4 h after N supply.

**Supplementary Figure S7.** Volcano plot visualizing the DEGs in B73.

**Supplementary Figure S8.** Venn diagram showing the overlap of 1,446 potential targets bound by ZmEREB97 in the promoter regions to the genes containing Motif 1 in the promoter regions from blue, dark orange, gray, and light cyan modules.

**Supplementary Figure S9.** Location of the Mu element, CRISPR/Cas9-edited site, and the *ZmEREB97* transcript levels in *zmereb97* mutants.

**Supplementary Figure S10.** Hierarchical cluster analysis of potential ZmEREB97-targeted genes (1,446 genes) in WT, *zmereb97-_MU_*, and *zmereb97_-CRI_* mutants.

**Supplementary Figure S11.** Analysis of *gene* expression and enzyme activity in WT and *zmereb97* mutants.

**Supplementary Table S1.** Primers used in this study.

**Supplementary Data Set 1.** Data of coexpression network analysis with WGCNA.

**Supplementary Data Set 2.** Data of motif prediction and TF identification.

**Supplementary Data Set 3.** Data of DEG analysis and GO classification.

## Dive Curated Terms

The following phenotypic, genotypic, and functional terms are of significance to the work described in this paper:

NLP7 Gramene: AT1G64530
NLP7 Araport: AT1G64530

SPL9 Gramene: At2g42200
SPL9 Araport: At2g42200

NIA1 Gramene: AT1G77760
NIA1 Araport: AT1G77760

NIA2 Gramene: AT1G37130
NIA2 Araport: AT1G37130

SPL Gramene: AT4G27330
SPL Araport: AT4G27330

nitrate CHEBI: CHEBI:17632

## References

[kiae277-B1] Agren G , Franklin O. Root: shoot ratios, optimization and nitrogen productivity. Ann Bot. 2003:92(6):795–800. 10.1093/aob/mcg203 14565938 PMC4243620

[kiae277-B2] Bartlett A , O’Malley RC, Huang S, Galli M, Nery JR, Gallavotti A, Ecker JR. Mapping genome-wide transcription-factor binding sites using DAP-seq. Nat Protoc. 2017:12(8):1659–1672. 10.1038/nprot.2017.055 28726847 PMC5576341

[kiae277-B3] Berger A , Boscari A, Horta AN, Maucourt M, Hanchi M, Bernillon S, Rolin D, Puppo A, Brouquisse R. Plant nitrate reductases regulate nitric oxide production and nitrogen-fixing metabolism during the *Medicago truncatula*–*Sinorhizobium meliloti* symbiosis. Front Plant Sci. 2020:11:1313. 10.3389/fpls.2020.01313 33013954 PMC7500168

[kiae277-B4] Cao H , Liu Z, Gao J, Jia Z, Shi Y, Kang K, Peng W, Wang Z, Chen L, Neuhaeuser B, et al ZmNRT1.1B (ZmNPF6.6) determines nitrogen use efficiency via regulation of nitrate transport and signalling in maize. Plant Biotechnol J. 2023:22(2):316–329. 10.1111/pbi.14185 37786281 PMC10826987

[kiae277-B5] Castaings L , Camargo A, Pocholle D, Gaudon V, Texier Y, Boutet-Mercey S, Taconnat L, Renou JP, Daniel-Vedele F, Fernandez E, et al The nodule inception-like protein 7 modulates nitrate sensing and metabolism in Arabidopsis. Plant J. 2009:57(3):426–435. 10.1111/j.1365-313X.2008.03695.x 18826430

[kiae277-B6] Chandler JW . Class VIIIb APETALA2 ethylene response factors in plant development. Trends in Plant Sci. 2018:23(2):151–162. 10.1016/j.tplants.2017.09.016 29074232

[kiae277-B7] Coen ES , Romero J, Doyle S, Elliott R, Murphy G, Carpenter R. Floricaula: a homeotic gene required for flower development in *Antirrhinum majus*. Cell. 1990:63(6):1311–1322. 10.1016/0092-8674(90)90426-F 1702033

[kiae277-B8] Fan X , Tang Z, Tan Y, Zhang Y, Luo B, Yang M, Lian X, Shen Q, Miler AJ, Xu G. Overexpression of a pH-sensitive nitrate transporter in rice increases crop yields. Proc Natl Acad Sci USA. 2016:113(26):7118–7123. 10.1073/pnas.1525184113 27274069 PMC4932942

[kiae277-B9] Feng H , Yan M, Fan X, Li B, Shen Q, Miller AJ, Xu G. Spatial expression and regulation of rice high-affinity nitrate transporters by nitrogen and carbon status. J Exp Bot. 2011:62(7):2319–2332. 10.1093/jxb/erq403 21220781

[kiae277-B10] Fischer RA , Byerlee D, Edmeades GO. Can technology deliver on the yield challenge to 2050? Presented at FAO Expert Meet. Rome.: How Feed World 2050; 2009.

[kiae277-B11] Fujimoto SY , Ohta M, Usui A, Shinshi H, Ohme-Takagi M. Arabidopsis ethylene-responsive element binding factors act as transcriptional activators or repressors of GCC box–mediated gene expression. Plant Cell. 2000:12(3):393–404. 10.1105/tpc.12.3.393 10715325 PMC139839

[kiae277-B12] Gan Y , Filleur S, Rahman A, Gotensparre S, Forde BG. Nutritional regulation of ANR1 and other root-expressed MADS-box genes in *Arabidopsis thaliana*. Planta. 2005:222(4):730–742. 10.1007/s00425-005-0020-3 16021502

[kiae277-B13] Gaudinier A , Rodriguez-Medina J, Zhang L, Olson A, Liseron-Monfils C, Bågman AM, Foret J, Abbitt S, Tang M, Li B, et al Transcriptional regulation of nitrogen-associated metabolism and growth. Nature. 2018:563(7730):259–264. 10.1038/s41586-018-0656-3 30356219

[kiae277-B14] Ge M , Wang Y, Liu Y, Jiang L, He B, Ning L, Du H, Lv Y, Zhou L, Lin F, et al The NIN-like protein 5 (ZmNLP5) transcription factor is involved in modulating the nitrogen response in maize. Plant J. 2020:102(2):353–368. 10.1111/tpj.14628 31793100 PMC7217196

[kiae277-B15] Gilkerson J , Tam R, Zhang A, Dreher K, Callis J. Cycloheximide assays to measure protein degradation in vivo in plants. Bio Protoc. 2016:6(17):e1919. 10.21769/BioProtoc.1919

[kiae277-B16] Glass ADM . Nitrogen use efficiency of crop plants: physiological constraints upon nitrogen absorption. Crit Rev Plant Sci. 2003:22(5):452–470. 10.1080/07352680390243512

[kiae277-B17] Good AG , Beatty PH. Fertilizing nature: a tragedy of excess in the commons. PLOS Biol. 2011:9(8):e1001124. 10.1371/journal.pbio.1001124 21857803 PMC3156687

[kiae277-B18] Guan P , Ripoll JJ, Wang R, Vuong L, Bailey-Steinitz LJ, Ye D, Crawford NM. Interacting TCP and NLP transcription factors control plant responses to nitrate availability. Proc Natl Acad Sci USA. 2017:114(9):2419–2424. 10.1073/pnas.1615676114 28202720 PMC5338533

[kiae277-B19] Guan P , Wang R, Nacry P, Breton G, Kay SA, Pruneda-Paz JL, Davani A, Crawford NM. Nitrate foraging by Arabidopsis roots is mediated by the transcription factor TCP20 through the systemic signaling pathway. Proc Natl Acad Sci USA. 2014:111(42):15267–15272. 10.1073/pnas.1411375111 25288754 PMC4210337

[kiae277-B20] Gutierrez RA , Stokes TL, Thum K, Xu X, Obertello M, Katari MS, Tanurdzic M, Dean A, Nero D, McClung CR, et al Systems approach identifies an organic nitrogen-responsive gene network that is regulated by the master clock control gene CCA1. Proc Natl Acad Sci USA. 2008:105(12):4939–4944. 10.1073/pnas.0800211105 18344319 PMC2290744

[kiae277-B21] Han M , Okamoto M, Beatty PH, Rothstein SJ, Good AG. The genetics of nitrogen use efficiency in crop plants. Annu Rev Genet. 2015:49(1):269–289. 10.1146/annurev-genet-112414-055037 26421509

[kiae277-B22] Hochholdinger F , Yu P, Marcon C. Genetic control of root system development in maize. Trends Plant Sci. 2017:23(1):79–88. 10.1016/j.tplants.2017.10.004 29170008

[kiae277-B23] Hu B , Wang W, Ou S, Tang J, Li H, Che R, Zhang Z, Chai X, Wang H, Wang Y, et al Variation in NRT1.1B contributes to nitrate-use divergence between rice subspecies. Nat Genet. 2015:47(7):834–838. 10.1038/ng.3337 26053497

[kiae277-B24] Jia L , Hu D, Wang J, Liang Y, Li F, Wang Y, Han Y. Genome-wide identification and functional analysis of nitrate transporter genes (*NPF*, *NRT2* and *NRT3*) in maize. Int J Mol Sci. 2023:24(16):12941. 10.3390/ijms241612941 37629121 PMC10454388

[kiae277-B25] Katayama H , Mori M, Kawamura Y, Tanaka T, Mori M, Hasegawa H. Production and characterization of transgenic rice plants carrying a high-affinity nitrate transporter gene (*OsNRT2.1*). Breeding Sci. 2009:59(3):237–243. 10.1270/jsbbs.59.237

[kiae277-B26] Kiba T , Inaba J, Kudo T, Ueda N, Konishi M, Mitsuda N, Takiguchi Y, Kondou Y, Yoshizumi T, Ohme-Takagi M, et al Repression of nitrogen starvation responses by members of the Arabidopsis GARP-type transcription factor NIGT1/HRS1 subfamily. Plant Cell. 2018:30(4):925–945. 10.1105/tpc.17.00810 29622567 PMC5969275

[kiae277-B27] Konishi M , Yanagisawa S. Arabidopsis NIN-like transcription factors have a central role in nitrate signalling. Nat Commun. 2013:4(1):1617. 10.1038/ncomms2621 23511481

[kiae277-B28] Krapp A . Plant nitrogen assimilation and its regulation: a complex puzzle with missing pieces. Curr Opin Plant Biol. 2015:25:115–122. 10.1016/j.pbi.2015.05.010 26037390

[kiae277-B29] Krouk G , Mirowski P, LeCun Y, Shasha DE, Coruzzi GM. Predictive network modeling of the high resolution dynamic plant transcriptome in response to nitrate. Genome Biol. 2010:11(12):123. 10.1186/gb-2010-11-12-r123 21182762 PMC3046483

[kiae277-B30] Leleu O , Vuylsteker C. Unusual regulatory nitrate reductase activity in cotyledons of *Brassica napus* seedlings: enhancement of nitrate reductase activity by ammonium supply. J Exp Bot. 2004:55(398):815–823. 10.1093/jxb/erh088 14990621

[kiae277-B31] Li B , Xin W, Sun S, Shen Q, Xu G. Physiological and molecular responses of nitrogen-starved rice plants to re-supply of different nitrogen sources. Plant Soil. 2006:287(1–2):145–159. 10.1007/s11104-006-9051-1

[kiae277-B32] Li S , Tian Y, Wu K, Ye Y, Yu J, Zhang J, Liu Q, Hu M, Li H, Tong Y, et al Modulating plant growth-metabolism coordination for sustainable agriculture. Nature. 2018:560(7720):595–600. 10.1038/s41586-018-0415-5 30111841 PMC6155485

[kiae277-B33] Liang L , Zhou L, Tang Y, Li N, Song T, Shao W, Zhang Z, Peng C, Feng F, Ma Y, et al A sequence-indexed mutator insertional library for maize functional genomics study. Plant Physiol. 2019:181(4):1404–1414. 10.1104/pp.19.00894 31636104 PMC6878021

[kiae277-B34] Lupini A , Mercati F, Araniti F, Miller AJ, Sunseri F, Abenavoli MR. NAR2.1/NRT2.1 functional interaction with NO_3_^−^and H^+^ fluxes in high-affinity nitrate transport in maize root regions. Plant Physiol Biochem. 2016:102:107–114. 10.1016/j.plaphy.2016.02.022 26926793

[kiae277-B35] Licausi F , Ohme-Takagi M, Perata P. APETALA2/Ethylene responsive Factor (AP2/ERF) transcription factors: mediators of stress responses and developmental programs. New Phytol. 2013:199(3):639–649. 10.1111/nph.12291 24010138

[kiae277-B36] Liu Y , Wang H, Jiang Z, Wang W, Xu R, Wang Q, Zhang Z, Li A, Liang Y, Ou S, et al Genomic basis of geographical adaptation to soil nitrogen in rice. Nature. 2021:590(7847):1–6. 10.1038/s41586-020-03091-w 33408412

[kiae277-B37] Los GV , Encell LP, McDougall MG, Hartzell DD, Karassina N, Zimprich C, Wood MG, Learish R, Ohana RF, Urh M, et al HaloTag: a novel protein labeling technology for cell imaging and protein analysis. ACS Chem Biol. 2008:3(6):373–382. 10.1021/cb800025k 18533659

[kiae277-B38] Machanick P , Bailey TL. MEME-ChIP: motif analysis of large DNA datasets. Bioinformatics. 2011:27(12):1696–1697. 10.1093/bioinformatics/btr189 21486936 PMC3106185

[kiae277-B39] Mahony S , Benos PV. STAMP: a web tool for exploring DNA-binding motif similarities. Nucleic Acids Res. 2007:35(Web Server):253–258. 10.1093/nar/gkm272 PMC1933206 17478497

[kiae277-B40] Marchive C , Roudier F, Castaings L, Bréhaut V, Blondet E, Colot V, Meyer C, Krapp A. Nuclear retention of the transcription factor NLP7 orchestrates the early response to nitrate in plants. Nat Commun. 2013:4(1):1713. 10.1038/ncomms2650 23591880

[kiae277-B41] Mikel MA , Dudley JW. Evolution of North American dent corn from public to proprietary germplasm. Crop Sci. 2006:46(3):1193–1205. 10.2135/cropsci2005.10-0371

[kiae277-B42] Mizoi J , Shinozaki K, Yamaguchi-Shinozaki K. AP2/ERF family transcription factors in plant abiotic stress responses. Biochem Biophys Acta. 2012:1819(2):86–96. 10.1016/j.bbagrm.2011.08.004 21867785

[kiae277-B43] Ning L , Wang J, Shi P, Wu C, Dong Z, Zhao H. Mapping genome-wide binding sites of endosperm specific expression transcription factor O2 using DAP-seq. Chinese Science Bulletin. 2019:64(24):2537–2548. 10.1360/N972019-00334

[kiae277-B44] O’Brien JA , Vega A, Bouguyon E, Krouk G, Gojon A, Coruzzi G, Gutiérrez RA. Nitrate transport, sensing, and responses in plants. Mol Plant. 2016:9(6):837–856. 10.1016/j.molp.2016.05.004 27212387

[kiae277-B45] Ort DR , Long SP. Limits on yields in the corn belt. Science. 2014:344:484–485. 10.1126/science.1253884 24786071

[kiae277-B46] Plett DC , Holtham LR, Okamoto M, Garnett TP. Nitrate uptake and its regulation in relation to improving nitrogen use efficiency in cereals. Semin Cell Dev Biol. 2018:74:97–104. 10.1016/j.semcdb.2017.08.027 28843981

[kiae277-B47] Robertson GP , Vitousek PM. Nitrogen in agriculture: balancing the cost of an essential resource. Annu Rev Environ Resour. 2009:34(1):97–125. 10.1146/annurev.environ.032108.105046

[kiae277-B48] Rose MD , Broach JR. Cloning genes by complementation in yeast. Methods Enzymol. 1991:194:195–230. 10.1016/0076-6879(91)94017-7 2005788

[kiae277-B49] Rubin G , Tohge T, Matsuda F, Saito K, Scheible WR. Members of the LBD family of transcription factors repress anthocyanin synthesis and affect additional nitrogen responses in Arabidopsis. Plant Cell. 2009:21(11):3567–3584. 10.1105/tpc.109.067041 19933203 PMC2798321

[kiae277-B50] Sakuraba Y , Chaganzhana MA, Iba K, Yanagisawa S. Enhanced *NRT1.1/NPF6.3* expression in shoots improves growth under nitrogen deficiency stress in Arabidopsis. Commun Biol. 2021:4(1):256. 10.1038/s42003-021-01775-1 33637855 PMC7910545

[kiae277-B51] Shoji T , Yuan L. ERF gene clusters: working together to regulate metabolism. Trends Plant Sci. 2021:26(1):23–32. 10.1016/j.tplants.2020.07.015 32883605

[kiae277-B52] Tang W , Ye J, Yao X, Zhao P, Xuan W, Tian Y, Zhang Y, Xu S, An H, Chen G, et al Genome-wide associated study identifies NAC42-activated nitrate transporter conferring high nitrogen use efficiency in rice. Nat Commu. 2019:10:5297. 10.1038/s41467-019-13187-1 PMC6872725 31754193

[kiae277-B53] Tang Z , Fan X, Li Q, Feng H, Miller AJ, Shen Q, Xu G. Knockdown of a rice stelar nitrate transporter alters long-distance translocation but not root influx. Plant Physiol. 2012:160(4):2052–2063. 10.1104/pp.112.204461 23093362 PMC3510131

[kiae277-B54] Thimm O , Bläsing O, Gibon Y, Nagel A, Meyer S, Krüger P, Selbig J, Müller LA, Rhee SY, Stitt M. MapMan: a user-driven tool to display genomics data sets onto diagrams of metabolic pathways and other biological processes. Plant J. 2004:37(6):914–939. 10.1111/j.1365-313X.2004.02016.x 14996223

[kiae277-B55] Trapnell C , Pachter L, Salzberg SL. Top Hat: discovering splice junctions with RNA-Seq. Bioinformatics. 2009:25(9):1105–1111. 10.1093/bioinformatics/btp120 19289445 PMC2672628

[kiae277-B56] Trevisan S , Borsa1 P, Botton A, Varotto S, Malagoli M, Ruperti B, Quaggiotti S. Expression of two maize putative nitrate transporters in response to nitrate and sugar availability. Plant Biol. 2008:10(4):462–475. 10.1111/j.1438-8677.2008.00041.x 18557906

[kiae277-B57] Tu X , Mejía-Guerra MK, Franco JAV, Tzeng D, Chu P, Shen W, Wei Y, Dai X, Li P, Buckler ES, et al Reconstructing the maize leaf regulatory network using ChIP-seq data of 104 transcription factors. Nat Commun. 2020:11(1):5089. 10.1038/s41467-020-18832-8 33037196 PMC7547689

[kiae277-B58] Vidal EA , Álvarez JM, Moyano TC, Gutiérrez RA. Transcriptional networks in the nitrate response of *Arabidopsis thaliana*. Curr Opin Plant Biol. 2015:27:125–132. 10.1016/j.pbi.2015.06.010 26247122

[kiae277-B59] Vitousek PM , Naylor R, Crews T, David MB, Drinkwater LE, Holland E, Johnes PJ, Katzenberger J, Martinelli LA, Matson PA. Nutrient imbalances in agricultural development. Science. 2009:324(5934):1519–1520. 10.1126/science.1170261 19541981

[kiae277-B60] Wang W , Hu B, Yuan D, Liu Y, Che R, Hu Y, Ou S, Liu Y, Zhang Z, Wang H, et al Expression of the nitrate transporter gene OsNRT1.1A/OsNPF6. 3 confers high yield and early maturation in rice. Plant Cell. 2018:30(3):638–651. 10.1105/tpc.17.00809 29475937 PMC5894839

[kiae277-B61] Wei S , Li X, Lu Z, Zhang H, Ye X, Zhou Y, Li J, Yan Y, Pei H, Duan F, et al A transcriptional regulator that boosts grain yields and shortens the growth duration of rice. Science. 2022:377(6604):386. 10.1126/science.abi8455 35862527

[kiae277-B62] Wen Z , Tyerman SD, Dechorgnat J, Ovchinnikova E, Dhugga KS, Kaiser BN. Maize NPF6 proteins are homologs of Arabidopsis CHL1 that are selective for both nitrate and chloride. Plant Cell. 2017:29(10):2581–2596. 10.1105/tpc.16.00724 28887406 PMC5774558

[kiae277-B63] Wu J , Lawit SJ, Weers B, Sun J, Mongar N, Hemert JV, Melo R, Meng X, Rupe M, Clapp J, et al Overexpression of *zmm28* increases maize grain yield in the field. Proc Natl Acad Sci USA. 2019:116(47):23850–23858. 10.1073/pnas.1902593116 31685622 PMC6876154

[kiae277-B64] Wu K , Wang S, Song W, Zhang J, Wang Y, Liu Q, Yu J, Ye Y, Li S, Chen J, et al Enhanced sustainable green revolution yield via nitrogen-responsive chromatin modulation in rice. Science. 2020:367(6478):eaaz2046. 10.1126/science.aaz2046 32029600

[kiae277-B65] Xu G , Fan X, Miller AJ. Plant nitrogen assimilation and use efficiency. Annu Rev Plant Biol. 2012:63(1):153–182. 10.1146/annurev-arplant-042811-105532 22224450

[kiae277-B66] Xuan W , Beeckman T, Xu G. Plant nitrogen nutrition: sensing and signaling. Curr Opin Plant Biol. 2017:39:57–65. 10.1016/j.pbi.2017.05.010 28614749

[kiae277-B67] Yamasaki K , Kigawa T, Seki M, Shinozaki K, Yokoyama S. DNA-binding domains of plant-specific transcription factors: structure, function, and evolution. Trends Plant Sci. 2012:5:267–276. 10.1016/j.tplants.2012.09.001 23040085

[kiae277-B68] Yan M , Fan X, Feng H, Miller AJ, Shen Q, Xu G. Rice OsNAR2.1 interacts with OsNRT2.1, OsNRT2.2 and OsNRT2.3a nitrate transporters to provide uptake over high and low concentration ranges. Plant Cell Environ. 2011:34(8):1360–1372. 10.1111/j.1365-3040.2011.02335.x 21486304

[kiae277-B69] Yilmaz A , Mejia-Guerra MK, Kurz K, Liang X, Welch L, Grotewold E. AGRIS: the Arabidopsis Gene Regulatory Information Server, an update. Nucleic Acids Res. 2011:39(Database):1118–1122. 10.1093/nar/gkq1120 PMC3013708 21059685

[kiae277-B70] Zhang G , Yi H, Gong J. The Arabidopsis ethylene/jasmonic acid-NRT signaling module coordinates nitrate reallocation and the trade-off between growth and environmental adaptation. Plant Cell. 2014:26(10):3984–3998. 10.1105/tpc.114.129296 25326291 PMC4247569

[kiae277-B71] Zhang H , Forde BG. An Arabidopsis MADS box gene that controls nutrient-induced changes in root architecture. Science. 1998:279(5349):407–409. 10.1126/science.279.5349.407 9430595

[kiae277-B72] Zhang Y , Liu T, Meyer CA, Eeckhoute J, Johnson DS, Bernstein BE, Nusbaum C, Myers RM, Brown M, Li W, et al Model-based analysis of ChIP-Seq (MACS). Genome Biol. 2008:9(9):R137. 10.1186/gb-2008-9-9-r137 18798982 PMC2592715

[kiae277-B73] Zhuang J , Deng D, Yao Q, Zhang J, Xiong F, Chen J, Xiong A. Discovery, phylogeny and expression patterns of AP2-like genes in maize. Plant Growth Regul. 2010:62(1):51–58. 10.1007/s10725-010-9484-7

