# Supplementary material for: Transcription factor ZmEREB97 regulates nitrate uptake in maize (Zea mays) roots
Source: Plant Physiol. 2024 May 14;196(1):535–50. doi: 10.1093/plphys/kiae277 (PMC11376383; doi:10.1093/plphys/kiae277)
Supplement: kiae277_Supplementary_Data [file kiae277_supplementary_data.zip › Supplementary Data.pdf]

Supplementary Table S1 Primers used in this study

Primer name	Sequence
ZmEREB97-F	TGACGCTGGAGCCGAGGTAC
ZmEREB97-R	GGCTCACGGCCAGCTTCTTG
ZmEREB97-Mu-F	GCGACGGGGAAGTTGGTCTTG
ZmEREB97-Mu-R	CATTGCTTCGGACGTGTGGAC
ZmEREB97-qPCR-F	CCAGCAAGCAGGCAGCATACTC
ZmEREB97-qPCR-R	GGCGGCGGCATGTTGAGG
T7-F	TAATACGACTCACTATAGGGAGATTATGAAGCTGACTGGCGTTGC
T7-R	TAATACGACTCACTATAGGGAGATGGAAGGTGTGTTCTGCGTTTC
ZmNRT1.1A-qPCR-F	CGTCGTCGGTCAGATTCATTGC
ZmNRT1.1A-qPCR-R	AGTGTCCCGAACGATCTTAGCC
ZmNRT1.1B-qPCR-F	TGAAGCAGGTGGTGCGGATG
ZmNRT1.1B-qPCR-R	GAGCAGGATGGAGCCGATGAG
ZmNRT1.2-qPCR-F	GCGGGATGCTGGAGGACTC
ZmNRT1.2-qPCR-R	CGAGGCGGTAGTGGTTCAGG
ZmNRT1.4A-qPCR-F	CGCCTCGCTCACCGTCTTC
ZmNRT1.4A-qPCR-R	CGACCCATGCCCTGCTACG
ZmNRT2.1-qPCR-F	ACGCTGCCGGTGGTGTTC
ZmNRT2.1-qPCR-R	TGCTCTTCTCGTCGTCGTTCC
ZmNRT2.5-qPCR-F	TGCTGCTTCCTCTCCACCTTC
ZmNRT2.5-qPCR-R	CGATGACGGCGGAGTAGTAGAC
ZmNRT2.7-qPCR-F	GGAGGCTGTGGCTTCTCTGG
ZmNRT2.7-qPCR-R	TGGACACGAACGGGACGATG
ZmNRT3.1A-qPCR-F	CGTCATCGAGACCCGCAAGAAG
ZmNRT3.1A-qPCR-R	GCACCAATGAATCAGCAACGACAG
ZmNRT3.1B-qPCR-F	AACGACGACTTGAGCAAGGAC
ZmNRT3.1B-qPCR-R	GCGACGTGGAAGTAGTAGGC
ZmNR1.1-qPCR-F	GACGCCGACCTCGCCAATG
ZmNR1.1-qPCR-R	CGTCGTGTCCTGGTGTGGTG
ZmNR1.1-qPCR-F	ATCCTCGCCTACATGCAGAACG
ZmNR1.1-qPCR-R	GACCCGCCTGAGCCACTTG
ZmGS1-qPCR-F	AGGGAAGACGACGGAGGAGAAG
ZmGS1-qPCR-R	GCACAGCACGCACGATGAAG
ZmGS2-qPCR-F	AACCTGCTCGTGAGTGTGCTG
ZmGS2-qPCR-F	GGTGTTGATGTCGGCGGTCTC
ZmUPF1-F	CACCCGGTTGGCTATGCTGTAC
ZmUPF1-R	TGTGCTCCACCAGAAGGCTGAC

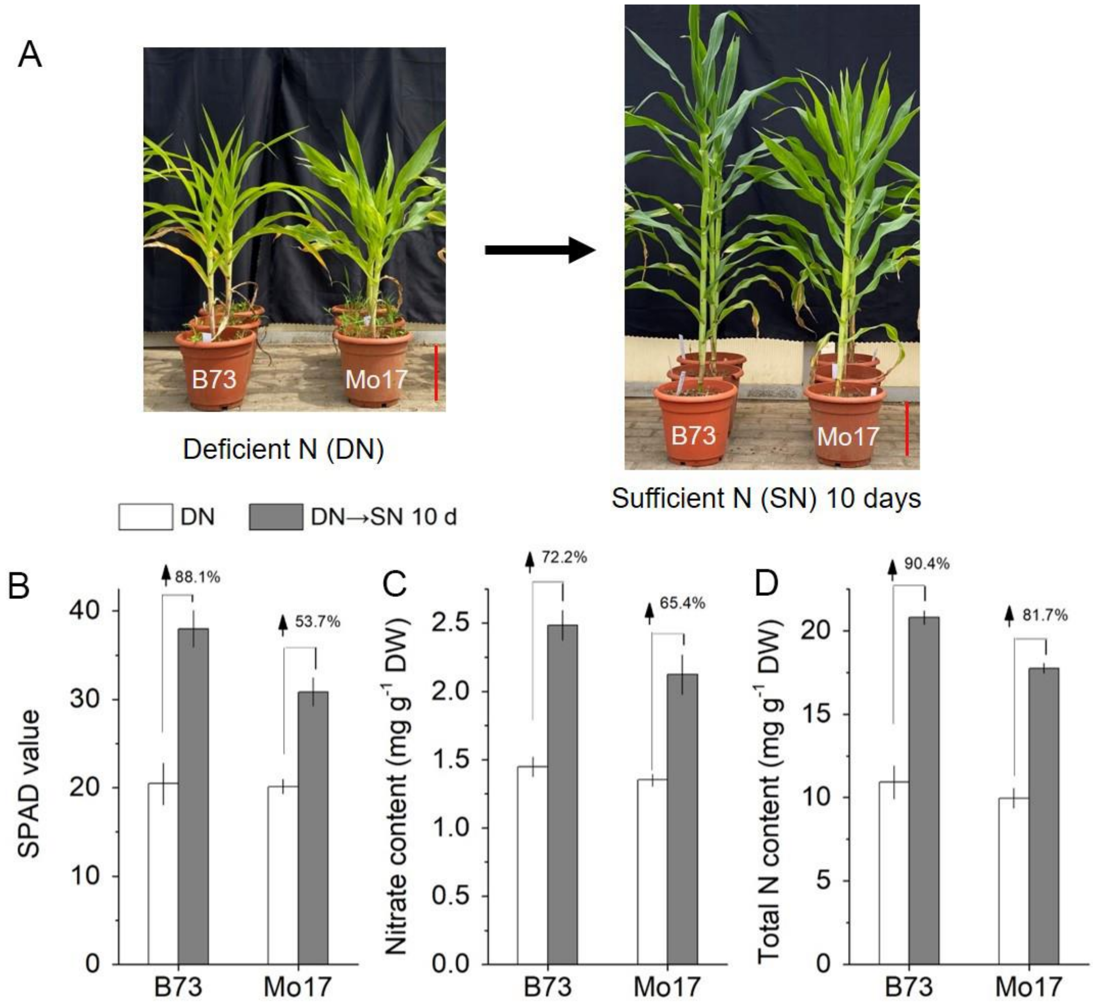

Supplementary Figure S1. Phenotypic and physiological analysis of B73 and Mo17 seedlings from deficient N condition to sufficient N condition. A, The phenotype of B73 and Mo17 seedlings. The B73 and Mo17 seedlings were pre-cultured in deficient N condition for 20 days, and then 10 mM KNO_3 solution was added. Bars = 30 cm. B-D, The SPAD value, nitrate and total N content in B73 and Mo17 seedlings. The error bars in represent means ± SD (n = 3).

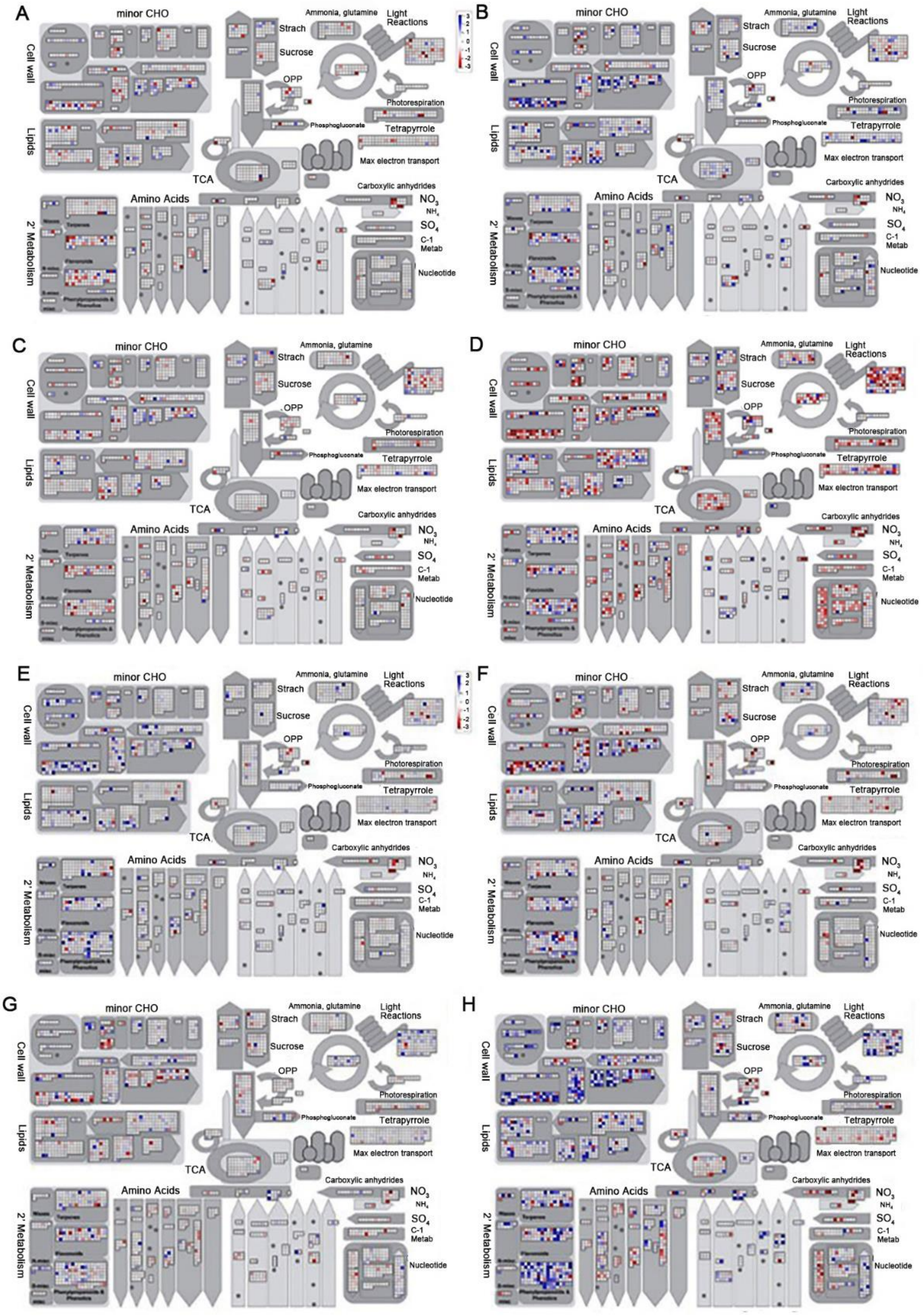

Supplementary Figure S2. Metabolism overview showing transcriptional profile during KCl and KNO_3 treatment at 0 hour and 8 hour in B73 and Mo17 root tissues. Log_2 ratios of FPKM were calculated and the resulting file was loaded onto MapMan to generate the overview. The B73 root tissues were treated with 15 mM KCl + 0.5 hour A, 15 mM KNO_3 + 0.5 hour. B, 15 mM KCl + 8 hour. C, 15 mM KNO_3 + 8 hour. D, respectively. The Mo17 root tissues were treated with 15 mM KCl + 0.5 hour. E, 15 mM KNO_3 + 0.5 hour. F, 15 mM KCl + 8 hour. G, 15 mM KNO_3 + 8 hour. H,

respectively.

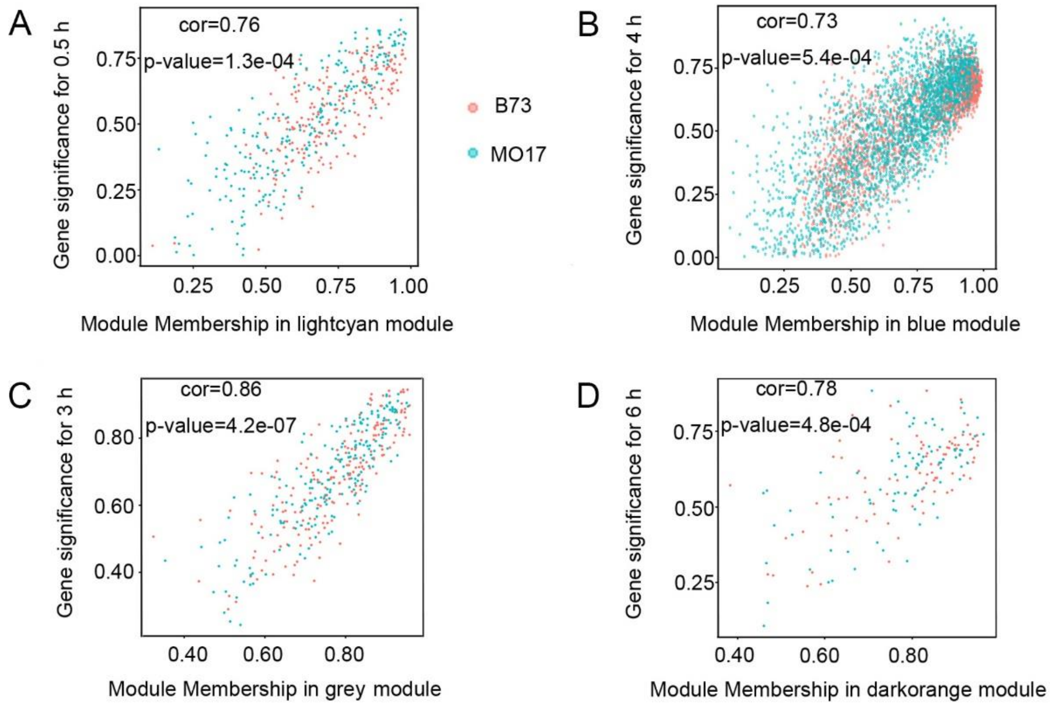

Supplementary Figure S3. Correlation scatter plots of Gene Significance (GS) and Module Membership (MM) in different modules. Gene Significance (GS) represents the correlation between gene expression and the nitrogen traits. Module Membership (MM) represents the correlation between gene and module characteristic values. A, 0.5 hours within the light cyan module. B, 4 hours within the blue module. C, 3 hours within the grey module. D, 6 hour within the dark orange module. Cor, corresponding correlation.

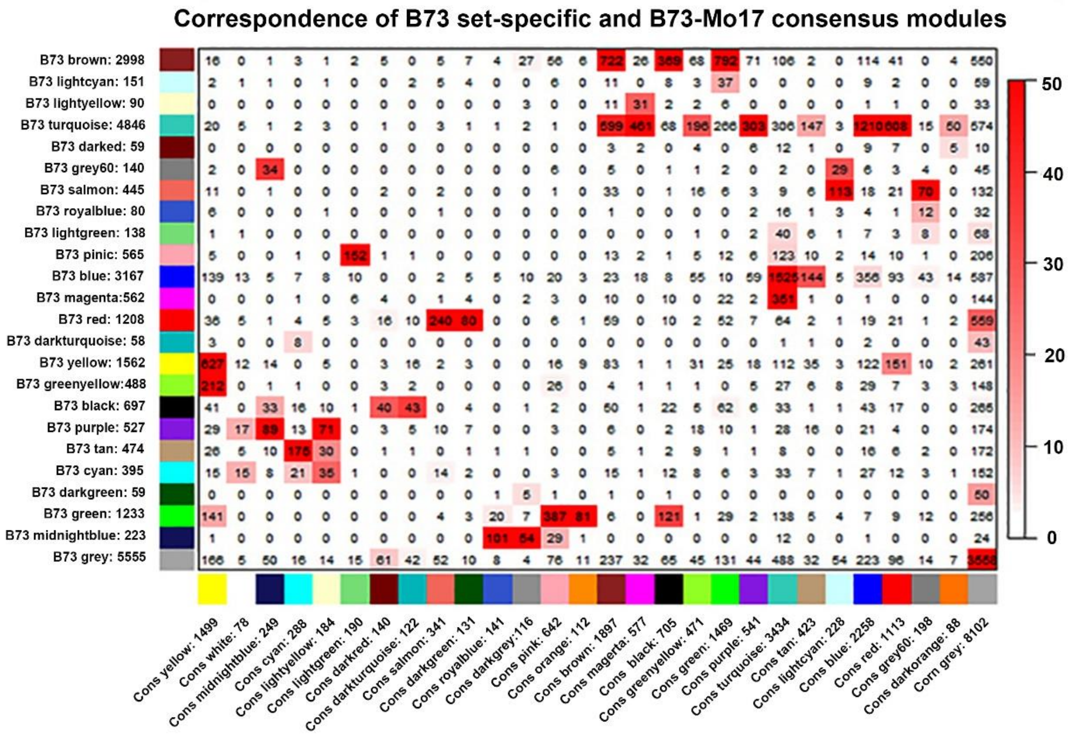

Supplementary Figure S4. Correspondence of B73 set-specific and B73-Mo17 consensus modules. Each row of the table corresponds to one B73 set-specific module, and each column corresponds to one consensus module. Numbers in the table indicate gene counts in the intersection of the corresponding modules. Coloring of the table encodes –log (p-value). The stronger the redcolor, the more significant is the overlap.

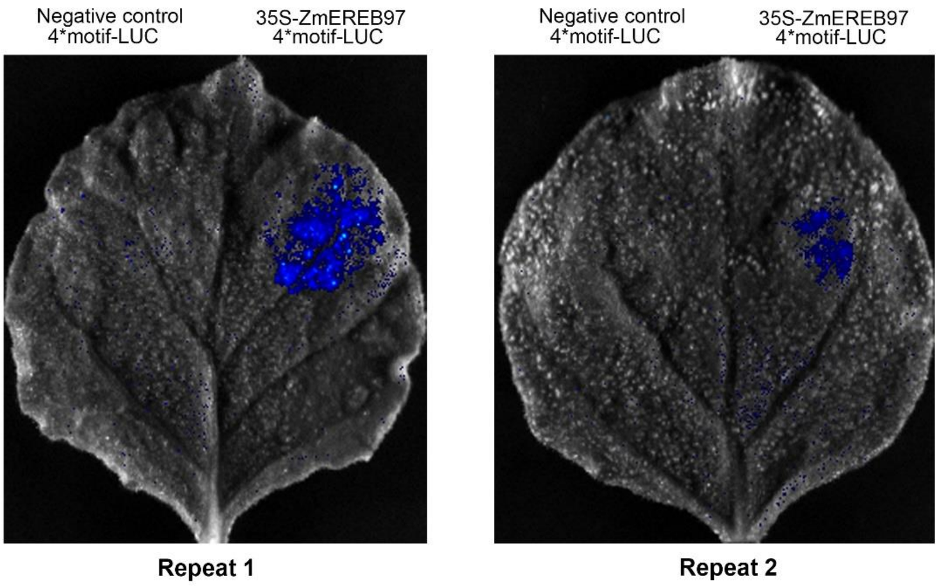

Supplementary Figure S5. Transcription activity assay of ZmEREB97 in *Nicotiana benthamiana* leaves.

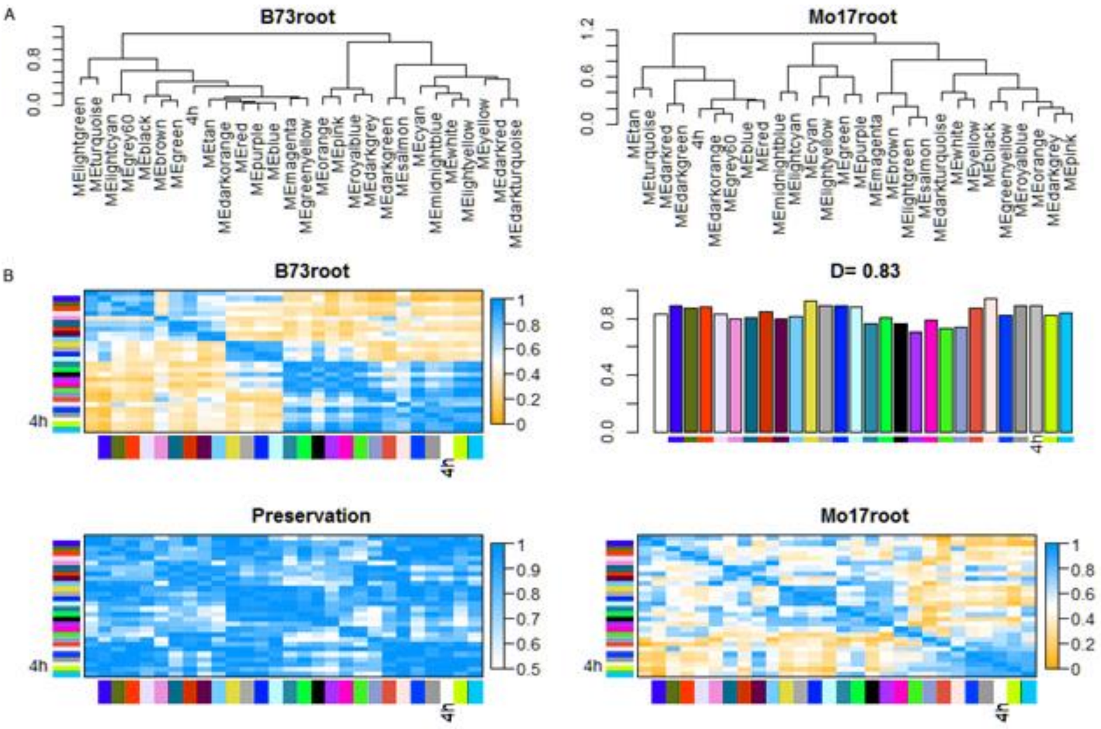

Supplementary Figure S6. Summary plot of consensus eigengene networks and their differential analysis in 4 hour after N supply. A, The dendrograms (clustering trees) of the consensus module eigengenes in B73 and Mo17 sets. B, Heatmap labeled eigengene networks and preservation networks. Blue, high adjacency (positive, correlation coefficient > 0.5); orange, low adjacency (negative, correlation coefficient < 0.5). Preservation network, one minus the absolute difference of the eigengene networks in the two data sets. The bar-plot depicts the column means of the preservation heatmap.

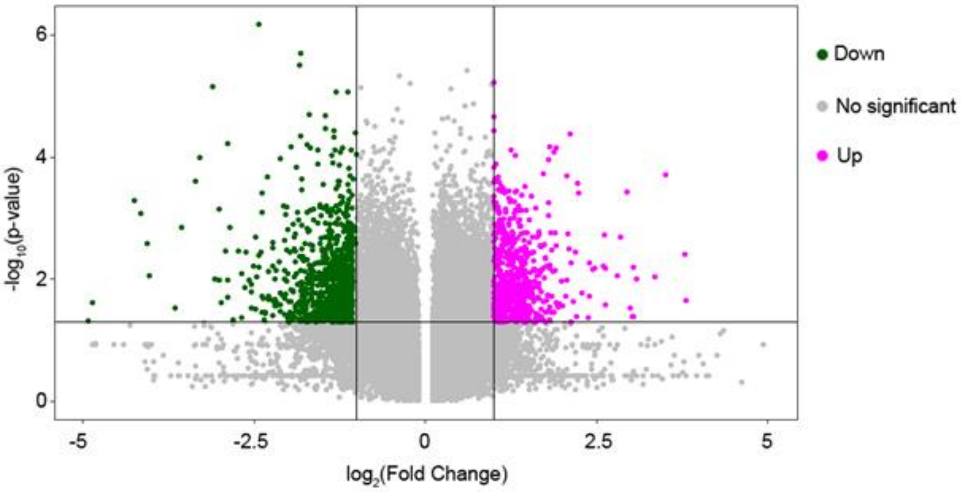

Supplementary Figure S7. Volcano plot visualizing the differentially expressed genes (DEGs) in B73. The X-axis represents the fold change in 15 mM KNO_3 vs. 15 mM KCl on a $\log_2$ scale. The y-axis represents the $-\log_{10}$ transformed p-values.

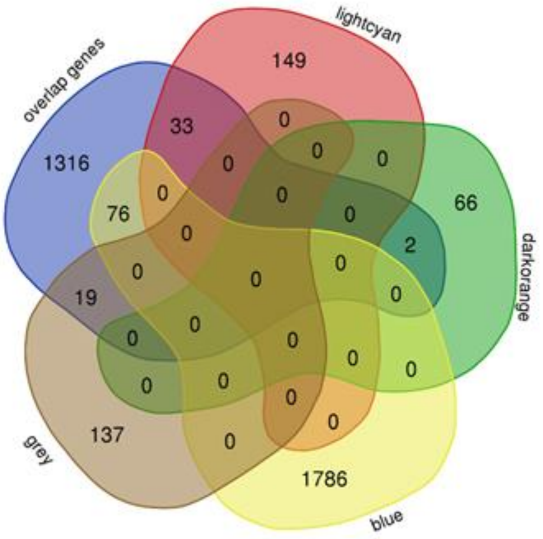

Supplementary Figure S8. Venn diagram showing the overlap of 1446 potential targets bound by ZmEREB97 in the promoter regions to the genes containing Motif 1 in the promoter regions from blue, dark-orange, grey and light-cyan module.

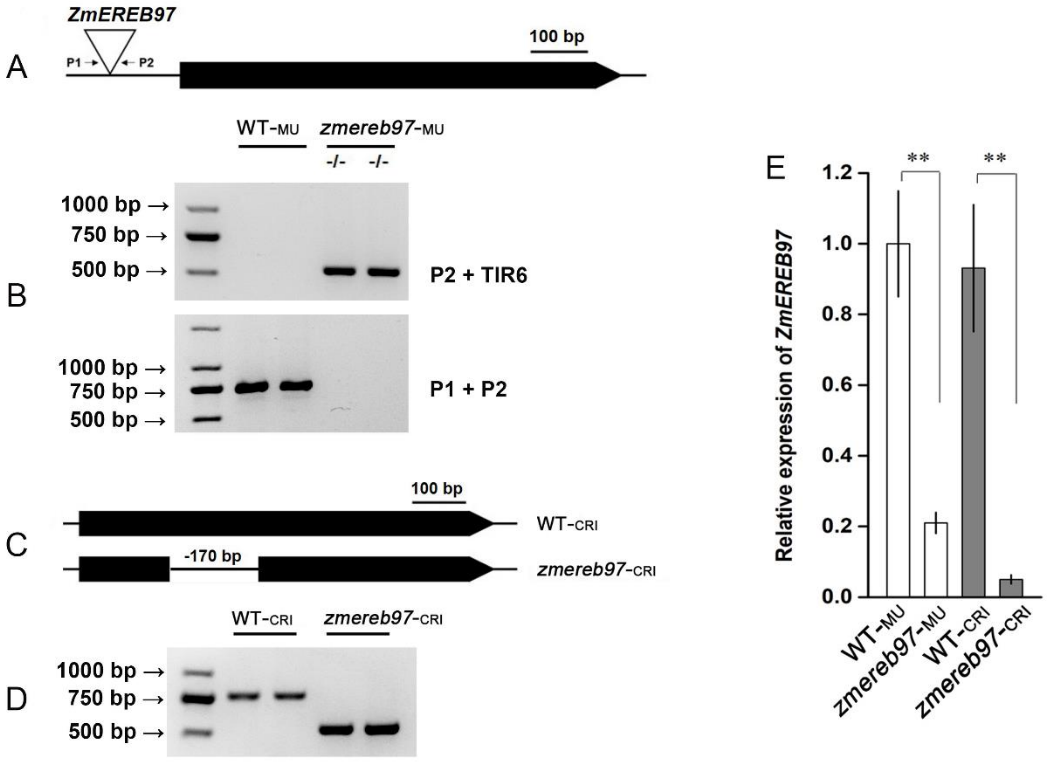

Supplementary Figure S9. Location of the Mu element, CRISPR/Cas9-edited site and the *ZmEREB97* transcript levels in *zmereb97* mutants. A, Gene structure of *ZmEREB97*. Boxes present exons. Location of the Mu element in *zmereb97* mutant is shown at triangle. B, Homozygous mutant plants were identified by the specific primers: P1 + P2 and P2 + TIR6. C to D, The CRISPR/Cas9-edited site in *zmereb97*-$_{\text{CRI}}$ mutant. *zmereb97*-$_{\text{CRI}}$ mutant has a 170 bp nucleotide deletion at in *zmereb97* coding sequence; E, *ZmEREB97* transcript levels in wild-type (WT), *zmereb97*-$_{\text{MU}}$ and *zmereb97*-$_{\text{CRI}}$ mutants by qPCR. The error bars in (C-J) and (M-O) represent means ± SD (n = 3). Lowercase letters indicate significant difference P < 0.01 according to Student's t-test.

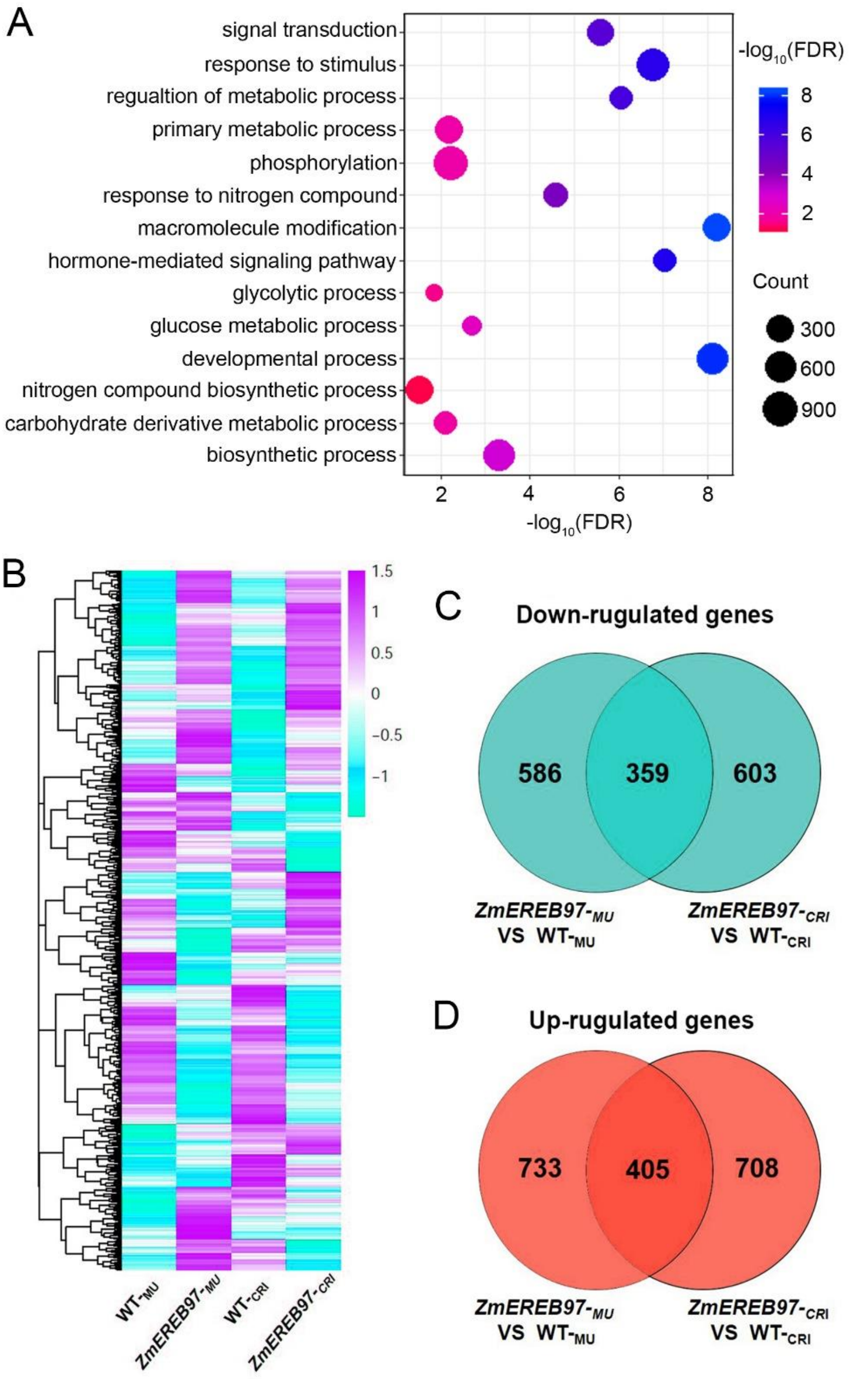

Supplementary Figure S10. Hierarchical cluster analysis of potential ZmEREB97-targeted genes (1446 genes) in WT, *zmereb97-MU* and *zmereb97-CRI* mutants. A, GO enrichment analysis of the 1,446 potential ZmEREB97-targeted genes. B, Clustering and Heatmap analysis of ZmEREB97-targeted genes. C to D, The Venn diagram showing the overlap gene numbers between WT and *zmereb97* mutants.

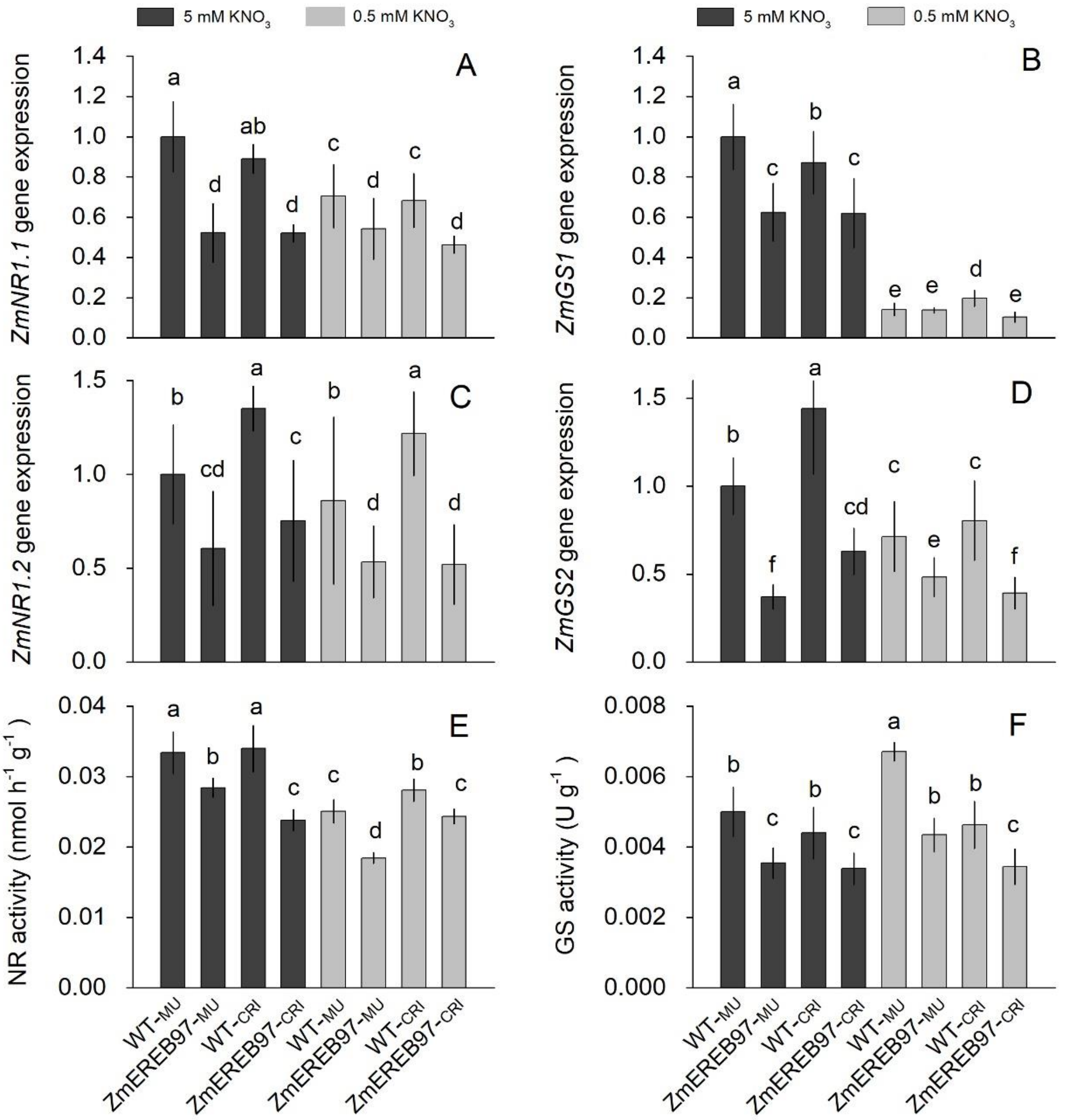

Supplementary Figure S11. Analysis of *gene* expression and enzyme activity in WT and *zmereb97* mutants. A-D, The expression of *ZmNR1.1*, *ZmNR1.2*, *ZmGS1*, *ZmGS2* in WT and *zmereb97* mutants on 5 mM and 0.5 mM KNO_3 treatments. E-F, The NR and GS activity in WT and *zmereb97* mutants on 5 mM and 0.5 mM KNO_3 treatments. The error bars in represent means ± SD (n = 3). Lowercase letters indicate significant difference at $P < 0.01$ according to Student's t-test (n = 3 collective sample each containing 3 biological replicates).
