# Supplementary material for: Transcription factor ZmEREB97 regulates nitrate uptake in maize (Zea mays) roots
Source: Plant Physiol. 2024 May 14;196(1):535–50. doi: 10.1093/plphys/kiae277 (PMC11376383; doi:10.1093/plphys/kiae277)
Supplement: kiae277_Supplementary_Data [file pp.00212.2024-s06.pdf › pp.00212.2024-s06.pdf]

Plant Physiology®

A novel AP2/ERF transcription factor, ZmEREB97, regulates nitrate uptake in maize (Zea mays L.) roots

Han Zhao, Qi Wu, Jin Xu, Yingdi Zhao, Yuancong Wang, Ling Zhou, Lihua Ning, and Sergey Shabala

Review Timeline:

Submission Date:	25-Apr-2022
Editorial Decision:	14-Jun-2022
Resubmission Received:	06-Feb-2024
Editorial Decision:	22-Feb-2024
Revision Received:	28-Mar-2024
Accepted:	12-Apr-2024

30-May-2022

Prof. Dr. Han Zhao
Institute of Crop Germplasm and Biotechnology, Provincial Key Laboratory of Agrobiology, Jiangsu Academy of Agricultural Sciences
Molecular breeding
Nanjing
China

RE: A novel AP2/ERF transcription factor, ZmEREB97, regulates nitrate uptake in maize (Zea mays L.) roots

Dear Dr. Han Zhao:

Thank you for giving us the opportunity to review your manuscript for publication in the Plant Physiology. The reviewers have found the manuscript of interest, but have raised a number of concerns that will require your attention. As these concerns are of some gravity and will entail substantial changes to the manuscript, you are likely to need additional time to address them. I must therefore reject the manuscript in its present form. Nonetheless, if you are able to address the points raised in review, I would welcome your resubmission of a manuscript that incorporates your responses.

In particular, I direct your attention to the following comments for consideration in a resubmitted manuscript.

Although both reviewers appreciate the novelty and importance of this finding, they identify as most critical point the in-planta validation of ZmEREB97 by using only one Mu-insertion line, which may have a very complex genetic background, potentially disturbing phenotypic analysis. At this point, a second insertion line or a complemented line is required to put the phenotypic characterization on solid ground.

Please note that any resubmission will be treated as a new entry to the system and is likely to go out for review.

If you do have any queries or concerns, you are welcome to contact me in the first instance.

Thank you again for allowing Plant Physiology to review your work. We look forward to hearing from you soon.

Sincerely,

Plant Physiology Board of Editors

-- Reviewer comments:
Reviewer #1 (Comments for the Author):

This study from Wu et al., reports on the identification of a transcription factor ZmEREB97 that functions as a positive regulator of nitrate acquisition in maize. By using combinatorial approaches of transcriptional network analysis, cis-element prediction and yeast one hybrid screening, the authors captured a AP2/ERF transcription factor ZmEREB97 that is involved in the nitrate signaling responses in maize. DAP-seq assay showed that ZmEREB97 is able to target to the promoter regions of many nitrate responsive genes in vitro. Functional characterization revealed that ZmEREB97 is required for the induction of several nitrate transporters upon nitrate supply, and thus it is critical for nitrate uptake in maize.

This study identifies a novel transcriptional regulator of nitrate uptake that not only uncovers the molecular mechanism of N acquisition in maize but also provides a putative genetic target to improving N use efficiency in crops, thus is an interesting progress for the scientific community. In addition, most experiments in this study are well conducted, and the main conclusions are justified.

However, thorough verifications on the role of ZmEREB97 as a potential key player in nitrate signaling pathway are still missing, which certainly limits the impact of this study. Major concerns are outlined below.

1. Since the morphological and physiological characterizations of ZmEREB97 was based on only one Mu insertion mutant (lack of the source information in materials and methods), it is very important to generate more zmereb97 mutants by CRISPR/Cas9 approach or construct ZmEREB97 complementation line in the background of zmereb97 mutant.
2. In Figure S7, several N-related metabolic processes were highlighted by the GO enrichment analysis among 1446 potential ZmEREB97-targeted genes, strongly indicating that ZmEREB97 may also play a key role in regulating N assimilation or metabolism. Therefore, more physiological parameters of nitrate assimilation and the expression of key enzymes of nitrate

metabolism need to be further tested in zmereb97 mutant upon differing N supplies.
3. Shown by Figure 8, the zmereb97 mutant seems to exhibit a constitutive N deficient symptom. It would be necessary to clarify whether the expression of some marker genes of N deficiency is induced in zmereb97 mutant even in the present of sufficient N supply. In addition, root-to-shoot ratio of biomass is a good indicator to evaluate the sensitivity of zmereb97 mutant to N supply.
4. To better verify the function of ZmEREB97 as a key player in nitrate signaling pathway, the expression of candidates among 1446 potential ZmEREB97-targeted genes need to be tested between wild type and zmereb97 mutant. Even better, a RNA-seq analysis between wild type and zmereb97 mutant will definitely provide a much deeper insight into the function of ZmEREB97 in nitrate signaling response.
5. It is still unclear how the six NRTs were selected in the experiment of Figure 10 to exam the influence of ZmEREB97 on nitrate uptake. Do they have GCC-box in their promoter region? Or do they belong to the potential ZmEREB97-targeted genes according to DAP-seq assay?
6. It is important to clarify whether manipulating the expression level of ZmEREB97 (by knocking down or over-expressing ZmEREB97) can really influence maize yield as well as N use efficiency in field condition, which will emphasize the bio-relevance of ZmEREB97 to the improvement of nutrient use efficiency.
7. Honestly, I am quite confused about the comparison of nitrate responses between maize inbred lines B73 and Mo17 at the beginning part of this story. I expect to see whether the nitrate response, nitrate uptake capacity, the induction or the activity of ZmEREB97 has any difference between B73 and Mo17 upon nitrate supply, but actually nothing was shown or discussed in the rest of the manuscript. If ZmEREB97-dependent nitrate signaling pathway has no different between B73 and Mo17, it makes no sense to present this comparison of transcriptome analysis of B73 and Mo17 only in Figure 1 but overlook at it at all in the rest part of the manuscript.
8. Instead of presenting a repetition of result section, the discussion needs to be largely redeveloped.

Reviewer #2 (Comments for the Author):

Wu et al have presented a promising manuscript detailing the selection of a N-related promoter element (AP2/ERF) using bioinformatic means, backed up with a Y1H assay to identify putative TF binding proteins. The selection of ZmEREB97 from the Y1H was further verified using transcriptional activation assays in tobacco with a luciferase activated marker, in situ PCR for tissue expression profiling, intercellular localisation (WB and mesophyll cell expression) to reveal a nuclear localisation and transcriptional expression assays to reveal a rapid N-responsiveness that declines within hours. A DAP-SEQ analysis identified ~1446 DEG's directly targeted by ZmEREB97, 130 of which belonging to the conserved motif 1 used to design bait targets in the Y1H assay. A mu transposon mutant was incorporated to help demonstrate functionality of the ZmEREB97. In general, the zmereb97 mutant displayed altered primary root length and later densities when grown at 10 mM KNO3 but not 2 mM KNO3. Shoot growth in the mutant was more dramatic (decreased) regardless of N supply. The mutant revealed a decrease in net nitrate flux around the roots, a decrease of flux into roots and over time a reduction in nitrate redistribution to the shoots. The change in nitrate transport was linked to altered expression profiles of several high and low affinity nitrate transport systems.

A few areas should be examined to further support these findings and suggested outcomes and role of ZmEREB97:

1) The use of a mu mutant maize line is exciting. However, the line's heritage and genetic pedigree to the WT being compared to needs further clarification. Has this line entered into a BC program to remove other MU transposons. Since the parentage of most MU populations is complex, an assurance that the mutant and the WT are comparable needs to be demonstrated. There is no reference or detailed information on the mutant's background.
2) The fact the TF has multiple targets in maize, the ability to directly link the TF to a specific N response remains difficult. Can the promoters of the identified nitrate transporter genes be tested in a Y1H assay to confirm physical interactions with the EREB97 TF?

Reply to reviewers

We thank the reviewers for their valuable comments. Below is a point-by-point response to the comments raised by the reviewers, and we have revised the manuscript accordingly.

Reviewer #1 (Comments for the Author):

This study from Wu et al., reports on the identification of a transcription factor ZmEREB97 that functions as a positive regulator of nitrate acquisition in maize. By using combinatorial approaches of transcriptional network analysis, cis-element prediction and yeast one hybrid screening, the authors captured a AP2/ERF transcription factor ZmEREB97 that is involved in the nitrate signaling responses in maize. DAP-seq assay showed that ZmEREB97 is able to target to the promoter regions of many nitrate responsive genes in vitro. Functional characterization revealed that ZmEREB97 is required for the induction of several nitrate transporters upon nitrate supply, and thus it is critical for nitrate uptake in maize.

This study identifies a novel transcriptional regulator of nitrate uptake that not only uncovers the molecular mechanism of N acquisition in maize but also provides a putative genetic target to improving N use efficiency in crops, thus is an interesting progress for the scientific community. In addition, most experiments in this study are well conducted, and the main conclusions are justified.

However, thorough verifications on the role of ZmEREB97 as a potential key player in nitrate signaling pathway are still missing, which certainly limits the impact of this study. Major concerns are outlined below.

1. Since the morphological and physiological characterizations of ZmEREB97 was based on only one Mu insertion mutant (lack of the source information in materials and methods), it is very important to generate more *zmereb97* mutants by CRISPR/Cas9 approach or construct ZmEREB97 complementation line in the background of *zmereb97* mutant.

Reply: Accepted. Following your recommendation, we have successfully developed a novel ZmEREB97 CRISPR-Cas9 transgenic line, as illustrated in Figure S9. This transgenic line was subsequently employed to help demonstrate the functionality of ZmEREB97. To ensure a thorough comprehension, we have included the background information and detailed specifics concerning the Mu insertion mutant (*zmereb97*-MU) and CRISPR/Cas9 mutant (*zmereb97*-CRI) in the M&M section (Line 550-562).

2. In Figure S7, several N-related metabolic processes were highlighted by the GO enrichment analysis among 1446 potential ZmEREB97-targeted genes, strongly indicating that ZmEREB97 may also play a key role in regulating N assimilation or metabolism. Therefore, more physiological parameters of nitrate assimilation and the expression of key enzymes of nitrate metabolism need to be further tested in *zmereb97* mutant upon differing N supplies.

Reply：Accepted. To address this concern, the revised version incorporates the results of testing two pivotal enzymes, NR and GS, implicated in nitrate assimilation. The expression and activity of these enzymes were assessed under varying nitrogen treatments (Line 369-386). The updated data is now presented in Fig. S11.

3. Shown by Figure 8, the zmereb97 mutant seems to exhibit a constitutive N deficient symptom. It would be necessary to clarify whether the expression of some marker genes of N deficiency is induced in zmereb97 mutant even in the present of sufficient N supply. In addition, root-to-shoot ratio of biomass is a good indicator to evaluate the sensitivity of zmereb97 mutant to N supply.

Reply: Accepted. In the revised version, we investigated the involvement of ZmEREB97 in NO_3^- signaling by examining the expression levels of NO_3^- response marker genes, specifically *ZmNR1.1*, *ZmNR1.2*, *ZmGS1*, and *ZmGS2* (Line 550-562). Our results reveal a significant reduction in the expression of these marker genes upon loss of ZmEREB97 function, irrespective of whether the nitrogen conditions are sufficient or deficient. Supplementary data supporting these findings are provided in Figure S11.

Additionally, we have included supplementary data in Figure 6E to assess the N sensitivity of the *zmereb97* mutants, with a specific focus on the root-to-shoot ratio as an indicator. Our findings indicate that the *zmereb97* mutants exhibit a higher root-to-shoot ratio, suggesting reduced sensitivity to N (Line 340-344).

4. To better verify the function of ZmEREB97 as a key player in nitrate signaling pathway, the expression of candidates among 1446 potential ZmEREB97-targeted genes need to be tested between wild type and *zmereb97* mutant. Even better, a RNA-seq analysis between wild type and *zmereb97* mutant will definitely provide a much deeper insight into the function of ZmEREB97 in nitrate signaling response.

Reply：To address this concern, we conducted an RNA-seq experiment with three biological replicates each for the WT and *zmereb97* mutants. Subsequently, we selected 1446 potential genes targeted by ZmEREB97 for further analysis. The results revealed that a total of 764 genes exhibited significant co-regulation (either up- or down-regulated) in both lines of *zmereb97* mutants, strongly suggesting a crucial role for ZmEREB97 in nitrate signaling response (Figure S10; Line 326-334).

5. It is still unclear how the six NRTs were selected in the experiment of Figure 10 to exam the influence of ZmEREB97 on nitrate uptake. Do they have GCC-box in their promoter region? Or do they belong to the potential ZmEREB97-targeted genes according to DAP-seq assay?

Reply：Accepted. Please disregard the initial six NRTs we selected. In the revised version, we conducted Y1H, qPCR, and dual-luciferase assays, ultimately identifying six authentic *NRT* genes that interact with ZmEREB97 (refer to Figures 8 and 9; Figures 10A-G; Line 387-415). EMSA confirmed the presence of GCC-box in the promoter region of all six *NRT* genes, demonstrating their recognition by the ZmEREB97 (Figure 10H-M, Line 416-423). Additionally, three interacted genes named *ZmNRT1.1A*,

ZmNRT2.1 and *ZmNRT2.5* were also included in the results of DAP-seq assay (Dataset S3).

6. It is important to clarify whether manipulating the expression level of ZmEREB97 (by knocking down or over-expressing ZmEREB97) can really influence maize yield as well as N use efficiency in field condition, which will emphasize the bio-relevance of ZmEREB97 to the improvement of nutrient use efficiency.

Reply： To address this concern, we conducted experiments under conditions approximating a field setting. The soil collected from the field underwent thorough mixing using a cement mixer to maintain a consistent soil environment. Supplementary data related to growth, biomass, nitrogen content, and grain yield have been included in Figure 6K-O to assess the potential contribution of ZmEREB97 to Nitrogen Use Efficiency (NUE) and N-dependent maize yield (Line 349-357).

7. Honestly, I am quite confused about the comparison of nitrate responses between maize inbred lines B73 and Mo17 at the beginning part of this story. I expect to see whether the nitrate response, nitrate uptake capacity, the induction or the activity of ZmEREB97 has any difference between B73 and Mo17 upon nitrate supply, but actually nothing was shown or discussed in the rest of the manuscript. If ZmEREB97-dependent nitrate signaling pathway has no different between B73 and Mo17, it makes no sense to present this comparison of transcriptome analysis of B73 and Mo17 only in Figure 1 but overlook at it at all in the rest part of the manuscript.

Reply: Good suggestion. In fact, we had conducted experiments on several maize inbred lines to assess their sensitivity to nitrate prior to this study. Among the selected lines, B73 and Mo17 exhibited significant differences in their response to nitrate (Line 152-161). Supplementary data supporting this finding is presented in Figure S1.

In this study, our objective is to identify conserved regulatory factors in the response to nitrate between B73 and Mo17. ZmEREB97 was identified from consensus modules derived from B73 and Mo17, serving as a conserved transcription factor involved in the nitrate response pathway.

8. Instead of presenting a repetition of result section, the discussion needs to be largely redeveloped.

Reply: Following your advice, the discussion section has undergone substantial revision to better emphasize the novelty and scientific rigor of this study (Line 426-545).

Reviewer #2 (Comments for the Author):

Wu et al have presented a promising manuscript detailing the selection of a N-related promoter element (AP2/ERF) using bioinformatic means, backed up with a Y1H assay to identify putative TF binding proteins. The selection of ZmEREB97 from the Y1H was further verified using transcriptional activation assays in tobacco with a luciferase activated marker, in situ PCR for tissue expression profiling, intercellular localisation

(WB and mesophyll cell expression) to reveal a nuclear localisation and transcriptional expression assays to reveal a rapid N-responsiveness that declines within hours. A DAP-SEQ analysis identified ~1446 DEG's directly targeted by ZmEREB97, 130 of which belonging to the conserved motif 1 used to design bait targets in the Y1H assay. A mu transposon mutant was incorporated to help demonstrate functionality of the ZmEREB97. In general, the zmereb97 mutant displayed altered primary root length and later densities when grown at 10 mM KNO_3 but not 2 mM KNO_3. Shoot growth in the mutant was more dramatic (decreased) regardless of N supply. The mutant revealed a decrease in net nitrate flux around the roots, a decrease of flux into roots and over time a reduction in nitrate redistribution to the shoots. The change in nitrate transport was linked to altered expression profiles of several high and low affinity nitrate transport systems.

A few areas should be examined to further support these findings and suggested outcomes and role of ZmEREB97:

1. The use of a mu mutant maize line is exciting. However, the line's heritage and genetic pedigree to the WT being compared to needs further clarification. Has this line entered into a BC program to remove other MU transposons. Since the parentage of most MU populations is complex, an assurance that the mutant and the WT are comparable needs to be demonstrated. There is no reference or detailed information on the mutant's background.

Reply: Thanks for your suggestion. The maize *zmereb97*-MU mutant in the B73 background was sourced from a Mu insertional library (Liang et al., 2019, Plant Physiology). The mutants underwent three generations of backcrossing to the B73 genetic background. Subsequently, both the wild-type (WT) and positive plants from a single corn cob were identified, and then self-pollinated to obtain homozygous seeds. The details and reference for the *zmereb97*-MU mutant have been incorporated into the Materials and Methods section in the revised version (Line 550-554).

2. The fact the TF has multiple targets in maize, the ability to directly link the TF to a specific N response remains difficult. Can the promoters of the identified nitrate transporter genes be tested in an Y1H assay to confirm physical interactions with the EREB97 TF?

Reply: Accepted. Following your guidance, we performed the Y1H assay in the revised version to validate potential interactions between the ZmEREB97 TF and 17 key nitrate transporter genes in maize. As shown in Figure 8, ZmEREB97 exhibited interactions with the promoters of nine *ZmNRT* genes (Line 387-393). Subsequently, we further confirmed six of these interactions through subsequent experiments including qPCR, EMSA, and dual-luciferase assay (Figures 9 and 10, Line 394-423). These findings provide additional evidence supporting the interactions between the ZmEREB97 TF and nitrate transporter genes.

22-Feb-2024

Prof. Dr. Han Zhao
Jiangsu Academy of Agricultural Sciences
Molecular breeding
Nanjing, Jiangsu
China

RE: A novel AP2/ERF transcription factor, ZmEREB97, regulates nitrate uptake in maize (Zea mays L.) roots

Dear Dr. Han Zhao:

We are pleased to accept your manuscript for publication in the Plant Physiology. This acceptance is contingent on revision based on the comments of our reviewers. In particular, please consider the comments made by Reviewer #3.

Please highlight all changes and include a detailed annotation to changes to the text, with line numbers, and noting your responses to the comments.

To submit your revised manuscript, click:

Link Not Available

If you cannot return the revised manuscript within 8 weeks of receipt, please let us know. Otherwise, we will assume that you have elected not to revise the manuscript and are withdrawing it.

Thank you for allowing us to review your work. We look forward to hearing from you soon.

Sincerely,

Plant Physiology Board of Editors

--- Reviewer comments:
Reviewer #1 (Comments for the Author):

The authors conducted several new experimental and made extensive revision. I am satisfied with their responses to my previous concerns. I do not have new comments.

Reviewer #3 (Comments for the Author):

The study from Wu et al. employed bioinformatic tools together with systemic Y1H screening to identify putative TF binding proteins that are critical for nitrate signaling and efficiency. They uncovered that a nitrate inducible AP2 family TF ZmEREB97 was able to bind GCC-box of many nitrate responsive genes and moreover targets six nitrate transporters for efficient nitrate acquisition. This is of great relevance for the maize growth and agronomical yield. Overall, the present study is experimentally well-done and would be interesting for a broad scientific community. However, there some minor points require further clarification.
1. Line 60, do these 1446 potential EREB97 target genes coming from 4 nitrate responsive modules? In the main text, it was

written that ...., including 130 motif1 carrying genes from four modules.
2. Line 161, authors showed some value changes for B73 and Mo17 between two N treatment, and then claimed that these changes are significantly different. Where are statistics to support the conclusion?
3. Line 111-113, the author wrote that the first TF ANR1 regulates the systemic nitrate repression. Obviously, this is definitely not justified, to my knowledge.
4. Line 648, authors claimed EREB97 confers specific nitrate response. Given that they have never tested transcriptional response to other N forms or N supply levels, other environmental stimuli as well, it would not justified to conclude that. In addition, these wordings appear also in other chapters.
5. Several grammatic errors, for example, largest groups in line 641.

Reply to reviewers

We thank the reviewers for their valuable comments. Below is a point-by-point response to the comments raised by the reviewers, and we have revised the manuscript accordingly. The modified details can be checked in Manuscript with changes highlighted

Reviewer #1 (Comments for the Author):

The authors conducted several new experimental and made extensive revision. I am satisfied with their responses to my previous concerns. I do not have new comments.

Reviewer #3 (Comments for the Author):

The study from Wu et al. employed bioinformatic tools together with systemic Y1H screening to identify putative TF binding proteins that are critical for nitrate signaling and efficiency. They uncovered that a nitrate inducible AP2 family TF ZmEREB97 was able to bind GCC-box of many nitrate responsive genes and moreover targets six nitrate transporters for efficient nitrate acquisition. This is of great relevance for the maize growth and agronomical yield. Overall, the present study is experimentally well-done and would be interesting for a broad scientific community. However, there some minor points require further clarification.

1. Line 60, do these 1446 potential EREB97 target genes coming from 4 nitrate responsive modules? In the main text, it was written that ...., including 130 motif1 carrying genes from four modules.

Reply: Thanks for your kindly remind. It is definitely incorrect point in Abstract section. The 1446 potential EREB97 target genes identified by the over overlapping results from DAP-seq assay and DEGs, it is obviously not from 4 nitrate responsive modules. We have corrected this mistake. Line 60.

2. Line 161, authors showed some value changes for B73 and Mo17 between two N treatment, and then claimed that these changes are significantly different. Where are statistics to support the conclusion?

Reply: In this study, our objective is to identify conserved regulatory factors in the response to nitrate between B73 and Mo17. Therefore, we chose two inbred maize lines with varying levels of nitrate sensitivity for experimentation. In order to better articulate our experiment, we have revised the description in the result section. Line 163-173

3. Line 111-113, the author wrote that the first TF ANR1 regulates the systemic nitrate repression. Obviously, this is definitely not justified, to my knowledge.

Reply: Accepted. We have modified this section to ensure its accuracy and scientifically. Line 112-114.

4. Line 448, authors claimed EREB97 confers specific nitrate response. Given that they have never tested transcriptional response to other N forms or N supply levels, other environmental stimuli as well, it would not justified to conclude that. In addition, these

wordings appear also in other chapters.
Reply: We strongly agree with your suggestion. Considering that we just conducted the time-course analysis of ZmEREB97 expression with 15 mM KNO_3, it is indeed not appropriate to use wording like "specify". We have corrected this section and also similar wording in other chapters. Line 432, 467

5. Several grammatic errors, for example, largest groups in line 461.
Reply: Accepted. All grammatical errors in the main text have been carefully reviewed and thoroughly corrected. Line 184, 231-236, 238, 247, 258, 326, 328, 333, 366, 415, 460.

12-Apr-2024
Prof. Dr. Han Zhao
Jiangsu Academy of Agricultural Sciences
Molecular breeding
Nanjing, Jiangsu
China

MSID: PP2024-RA-00212DR1
MS TITLE: A novel AP2/ERF transcription factor, ZmEREB97, regulates nitrate uptake in maize (Zea mays L.) roots

Dear Dr. Han Zhao:

I am pleased to inform you that your revised manuscript "A novel AP2/ERF transcription factor, ZmEREB97, regulates nitrate uptake in maize (Zea mays L.) roots" has been accepted for publication in Plant Physiology. Your manuscript will be checked for consistency with journal formatting and image standards by one of our Science Editors, who will contact you shortly. They may have a number of queries and suggestions for you. Please keep in mind that they work to increase the online visibility of your article and its attraction for readers from the widest of backgrounds. You will have the opportunity to review any revisions, and I hope you will work constructively with them to get the most out of your research. You will also have the opportunity to review all changes when you receive the author proofs for your article.

Once validated for publication, your article will be sent to a Science Editor for a final review of your manuscript and source files. After your final files are uploaded, your article will appear in the next available issue and the online Preview version of your article will be posted after your signed Copyright and Checklist forms and final source files are received.

Please be sure to include the following in your manuscript file:
Harvard style citations
List of all author contributions and funding information at the beginning of manuscript after the titles and author list.
List all supplemental materials associated with the article, with titles, at the end of the ms, before the references and acknowledgments section.
Complete Figure Legends for all figures appearing in the article listed before the Literature Cited.

Thank you and congratulations on your Plant Physiology paper!

Sincerely,
Plant Physiology Board of Editors

=====
FIRST AUTHOR PROFILES
=====

We recognize the hard work that goes into being First Author of a Plant Physiology paper by publishing a "First Author Feature" for each First Author alongside your paper.

If the first author(s) would like to submit information for a feature on Facebook and Twitter, fill out the form by clicking on the link below:

Plant Physiology First Author Feature Form: https://forms.office.com/r/ZBCGZfhm8F
If you cannot access the form through the link above, please contact Rachel at rbelsky@aspb.org.

To upload an image of yourself to accompany your profile, please put your photo in the correct journal folder at the link below. You must label your image: Author First Name Last Name Article Title.

First Author Profile Image Dropbox: https://www.dropbox.com/scl/fo/xz029p7qjr2jml2rp2kmq/h?dl=0&rlkey=6fkxcaqmxx35ap152b2osy1fz

=====

IMPORTANT REMINDER: PEER REVIEW REPORTS
=====

If you opted to publish a peer review report along with your article during the original submission process, it will be prepared by the editorial staff and publicly posted with your manuscript, inside the zip file that contains any other supplemental material. As a reminder, the peer review report is a public record of all comments from editors and reviewers, as well as your prior responses, as you received them in the decision letters for each draft of your manuscript. If you agreed to publish this report and have changed your mind, or are not sure if you selected this option, please contact the editorial office as soon as possible before signing the license agreement from our publisher.

-- Reviewer comments:
Reviewer #4 (Comments for the Author):

The authors have fullly reivised the ms and clarified my concerns. I have no further comments and look forward to seeing the online ms soon.
